# TGF-β1-SOX9 axis-inducible COL10A1 promotes invasion and metastasis in gastric cancer via epithelial-to-mesenchymal transition

**DOI:** 10.1038/s41419-018-0877-2

**Published:** 2018-08-28

**Authors:** Tingting Li, Haipeng Huang, Guangyao Shi, Liying Zhao, Tuanjie Li, Ze Zhang, Ruoyan Liu, Yanfeng Hu, Hao Liu, Jiang Yu, Guoxin Li

**Affiliations:** 1 Department of General Surgery, Nanfang Hospital, Southern Medical University, Guangzhou, China; 2 Department of Gastrointestinal Surgery, Guangdong Provincial Hospital of Traditional Chinese Medicine, Guangzhou, China; 3 Division of Cardiology, Third Affiliated Hospital, Sun Yat-sen University, Guangzhou, China; 4 Departments of Maxillofacial and Otorhinolaryngology Oncology; Department of Head and Neck Surgery, Tianjin Medical University Cancer Institute and Hospital; National Clinical Research Center for Cancer, Key Laboratory of Cancer Prevention and Therapy, Tianjin; Tianjin’s Clinical Research Center for Cancer, Tianjin, China; 5 Department of Gynecologic Oncology, Tianjin Medical University Cancer Institute and Hospital; National Clinical Research Center for Cancer, Key Laboratory of Cancer Prevention and Therapy, Tianjin, Tianjin’s Clinical Research Center for Cancer, Tianjin, China

## Abstract

Molecular biomarkers that predict disease progression might promote drug development and therapeutic strategies in aggressive cancers, such as gastric cancer (GC). High-throughput mRNA sequencing (RNA-seq) revealed that collagen type X alpha 1 (COL10A1) is a disease progression-associated gene. Analysis of 103 GC patients showed that high COL10A1 mRNA expression was associated with GC metastasis and reduced survival. We analyzed the COL10A1 promoter using the UCSC genome website and JASPAR database, and we found potential SOX9 binding site. Here, we demonstrated that SOX9 and COL10A1 were both up-regulated in GC. We observed a positive correlation between the expression patterns of SOX9 and COL10A1 in GC cells and tissues. The results of electrophoretic mobility shift assay (EMSA), chromatin immunoprecipitation (ChIP) assay and promoter reporter indicated that SOX9 could directly bind to the COL10A1 gene promoter and activate its transcription. Biological function experiments showed that COL10A1 regulated the migration and invasion of GC cells. Knockdown COL10A1 inhibited lung and abdominal cavity metastasis in a nude mouse model. Moreover, transforming growth factor-β1 (TGF-β1) treatment up-regulated the phosphorylation of Smad2 and increased SOX9 and COL10A1 expression. COL10A1 was confirmed to be a potential inducer of epithelial-to-mesenchymal transition (EMT). SOX9 was essential for COL10A1-mediated EMT, and cell migration, invasion and metastasis. Co-expression of SOX9 and COL10A1 was associated with tumor progression and was strongly predictive of overall survival in GC patients. In summary, this study elucidated the mechanistic link between COL10A1 and the TGF-β1-SOX9 axis. These findings indicated that COL10A1 might play a crucial role in GC progression and serve as a potential biomarker and therapeutic target in GC patients.

## Introduction

Despite advancements of treatment over recent decades, gastric cancer (GC) remains one of the most common cancers, with a high incidence and large number of deaths worldwide^1^. The most frequent pattern of recurrence and metastasis in GC patients is peritoneal carcinomatosis (PC)^2^. An essential event in cancer cell metastasis is increasing migration and invasion. Many changes are required for this critical step, including loosening of the tight cell-cell adhesions between epithelial cancer cells and extracellular matrix (ECM) and cells, and degrading adjacent tissues by the matrix metalloproteinases (MMP)-2 and 9^3–6^. The epithelial-mesenchymal transition (EMT) can also enhance tumor progression and metastasis^7–10^. Therefore, there is an urgent need to identify the metastatic markers and understand the disease-progression patterns and molecular mechanisms of GC invasion and progression^11,12^.

In this study, we analyzed the RNA-req of tissue samples obtained after surgical resection of three early stage and three advanced stage GC patients with PC to identify markers that were correlated with advanced and metastatic disease and poor prognosis. Among the most highly expressed 10 genes both in the Stage I and Stage IV (Table 1), Type X collagen gene (COL10A1) was the gene with the second highest expression level. COL10A1, a secreted, short-chain collagen, belongs to the collagen family, which is a major interstitial matrix component. The ECM has been identified to play a critical role in a number of fundamental biological processes, such as cell differentiation, morphogenesis, growth, apoptosis and migration^13,14^. Tissue cancer cells can feel and respond to the stiffness of the local matrix in processes of development, differentiation, disease and regeneration^15^. Several reports have analyzed the differential gene expression profiles of normal and neoplastic tissue of different cancers and identified a set of genes including COL10A1^16–18^. Overexpression of COL10A1 protein levels correlates with colon cancer^19,20^. However, the pathophysiological role and relevance of COL10A1 to GC invasion and metastasis remain unknown.

**Table 1:** The 10 up and 5 down-regulated genes in the EGC and AGC groups

Gene symbol	Cancer(FPKM)	Normal(FPKM)	F.D.R
CST1	114.5467	0.83	5.54E-06
COL10A1	12.23667	0.103333	2.90E-05
ESM1	8.11	0.186667	1.13E-06
COL11A1	2.073333	0.05	0.00023
HOXC8	1.51	0.043333	0.024446
THBS4	19.48333	0.583333	0.005149
KLK6	5.09	0.156667	0.001224
SFRP4	16.4	0.55	8.31E-07
BAAT	1.883333	0.063333	0.00793
CST2	15.29	0.613333	0.00015
APOC3	0.001	17.41	0.006956
TMPRSS15	0.076667	28.62667	0.003162
OTOP3	0.04	5.976667	0.014279
APOB	0.093333	7.44	0.029445
CYP3A4	0.633333	24.80333	0.048529

SOX9, known as the high mobility group box transcription factor, is overexpressed in colorectal, prostate, and lung cancers^21–24^. He et al^25^. have reported that SOX9 and AP-1 family members cooperate to control chondrocyte hypertrophy through the activation of a COL10A1 enhancer. However, Leung et al^26^. have reported that forced ectopic SOX9 expression in chondrocyte hypertrophy results in a down-regulation of COL10A1 in vitro and in vivo. SOX9 transcriptional regulation of COL10A1 expression remains controversial. Furthermore, the mechanism for regulating the transcriptional activity of COL10A1 by SOX9 to promote GC cell EMT, migration, invasion and metastasis has not been elucidated.

In the present study, we identified and validated the role of COL10A1 in the migration and invasion of gastric cancer cells. Moreover, COL10A1 expression significantly correlated with poor clinical outcomes for GC patients. We provided direct functional evidence that COL10A1 overexpression enhanced EMT, which was induced by TGF-β1 and promoted invasiveness and disease progression that was directly regulated by SOX9. Taken together, the molecular mechanisms underlying COL10A1-enhanced cancer cell invasion were elucidated, providing an understanding of novel diagnostic and therapeutic strategies.

## Result

### RNA-seq analysis of GC reveals different gene expression profiles between early stage and advanced stage patients and identifies elevated COL10A1 levels on pathogenesis and progression

**Fig. 1:**
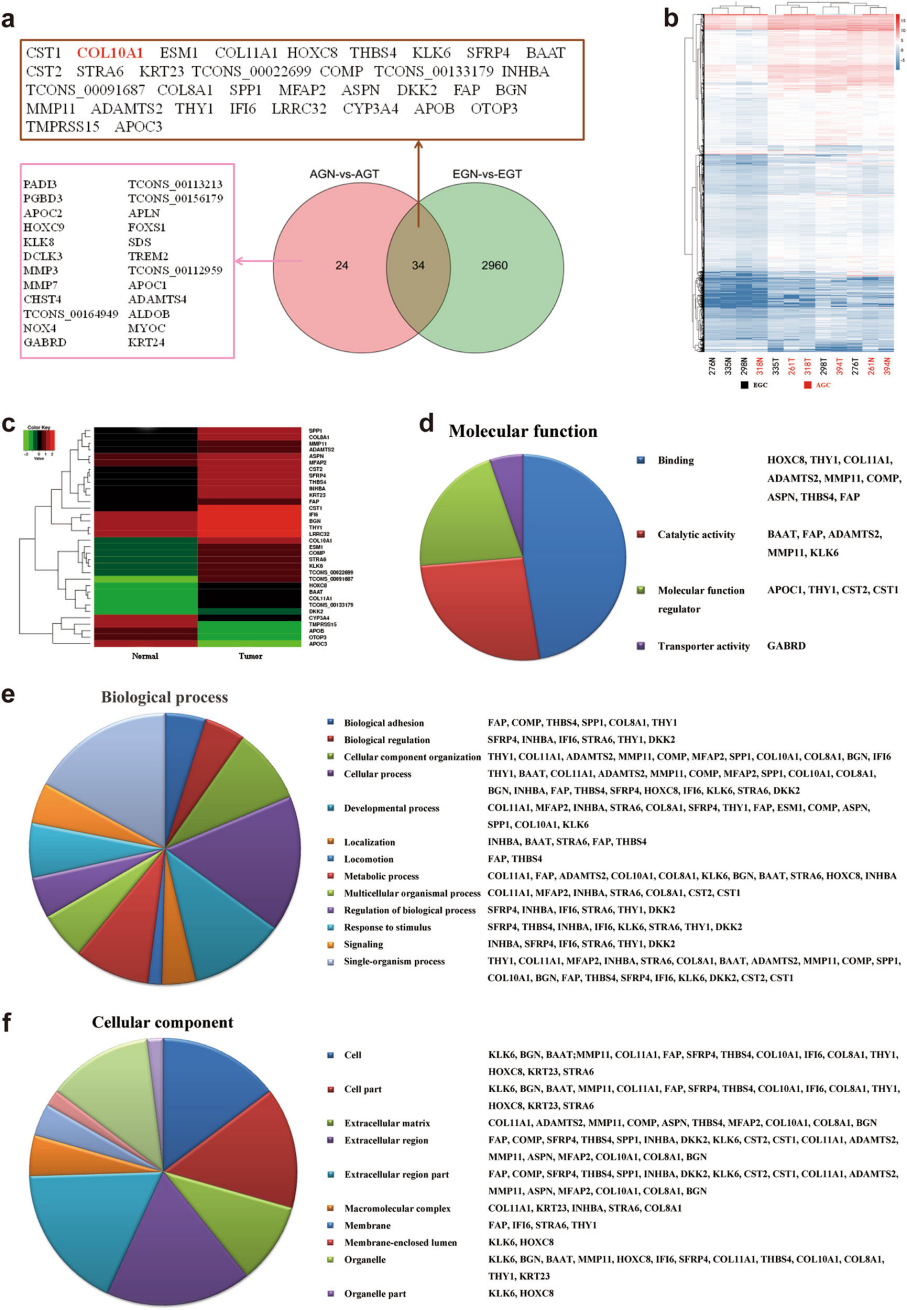
The difference in identified DEGs in the tumor compared with the normal phenotype. **a** Venn diagram of DEGs in different tumor stages. EGN and EGT represented Stage I normal and tumor groups, AGN and AGT represented Stage IV normal and tumor groups. **b** Hierarchical clustering of 12 samples using all DEGs. The number of EGC samples (black) and AGC samples (red) are indicated below heatmap. **c** Heat-map analysis of the 34 DEGs that were differentially expressed between the normal and tumor groups both in the EGC and AGC groups. Individual genes are arranged in rows and groups in columns. **d**, **e**, **f** Pie chart representation of the Gene Ontology (GO) annotations for DGEs in RNA-req and summarized according to molecular function, biological process and cellular component, among the 26 up-regulated genes both in the EGC and AGC groups, respectively

The transcriptomes was profiled by RNA-seq in six GC patients, three early stage gastric cancer (EGC) and three advanced stage gastric cancer (AGC). Complete patient characteristics and clinical-pathological parameters are shown in Supplementary Table 3. In total, 236.5 and 250.2 million sequencing reads were obtained in normal and cancer tissues in EGC group, as compared with 231.9 and 229.0 million in AGC group (Supplementary Table 4). A threshold FDR < 0.05 resulted in 34 significantly differentially expressed genes between normal and tumor samples both in EGC group and AGC group. The top 10 up and 5 down-regulated genes are listed in Table 1. To investigate the overlaps among these sets of DEGs, we used a Venn diagram to show the results (Fig. 1a). Detailed results of DEGs in each group are shown in Supplementary Tables 5 and 6. Heat maps of all DEGs and the 34 DEGs both in EGC group and AGC group are depicted in Figs. 1b, c.

The Database for Annotation, Visualization and Integrated Discovery was performed for Gene ontology analysis of the 26 up-regulated genes (excluding 3 unknown genes) both in EGC group and AGC group. The major molecular function, biological process and cellular component of the 26 genes were found to be associated with protein binding and bridging, cellular process and extracellular region, respectively (Fig. 1d, e, f). Notably, COL10A1 ranked as the gene with second highest fold-change and might be associated with disease pathogenesis and progression of GC. Therefore, COL10A1 was selected for systematic experimental analysis.

### mRNA expression and prognostic significance of COL10A1 in GC patients

**Fig. 2:**
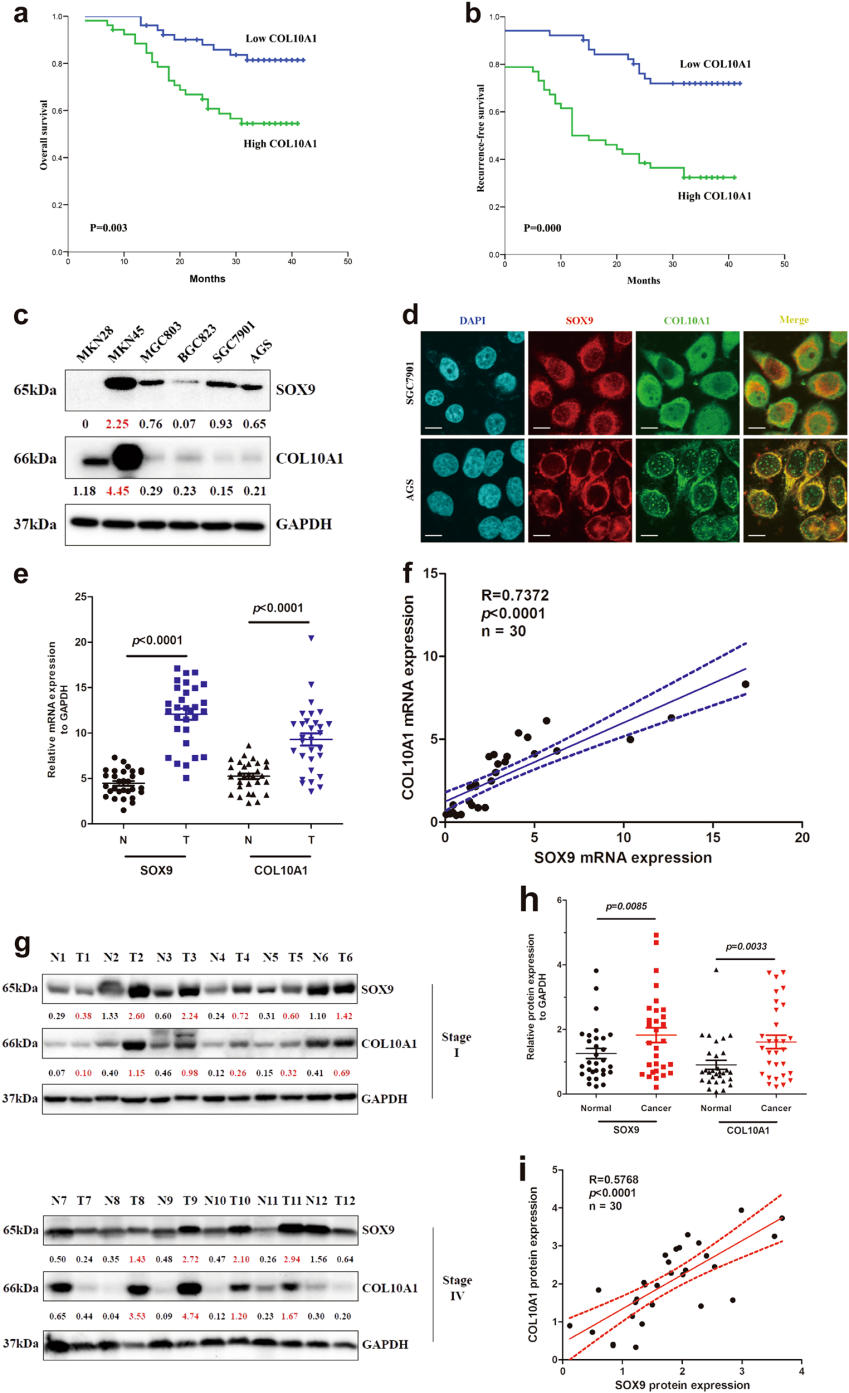
The prognostic value of COL10A1 mRNA in OS and RFS, and the expression of SOX9 and COL10A1 in cell lines and GC tissues. **a**, **b** Kaplan–Meier curves and log-rank test showing the prognostic value of COL10A1 mRNA in OS and RFS in 103 gastric cancer patients. **c** SOX9 and COL10A1 proteins were detected in GC cell lines by western blot analysis. **d** SOX9 and COL10A1 were localized by confocal immunofluorescence microscopy in the SGC7901 and AGS cell lines. **e**, **f** SOX9 and COL10A1 mRNA expression levels in normal and GC tissues. The mRNA expression levels of SOX9 and COL10A1 were semi-quantified, and the correlation between these two genes was evaluated with Spearman’s rank correlation method. **g** Proteins isolated from normal and GC tissues were analyzed by western blot analysis of SOX9 and COL10A1. The upper panel represents Stage I normal and tumor specimens; the lower panel represents Stage IV normal and tumor specimens. **h** and **i** SOX9 and COL10A1 protein expression levels in normal and GC tissues. The protein expression levels of SOX9 and COL10A1 were semi-quantified, and the correlations between these two genes were evaluated with Spearman’s rank correlation method. Scale bars represent 10 μm in (**d**)

We next analyzed COL10A1 mRNA expression levels in 103 GC patients by QPCR. The COL10A1 mRNA expression levels in 103 GC patients ranged from 1.51 to 20.41. A cut-off value (8.08) through ROC analysis (Supplementary Fig. 2a) was used to categorize the tumors into groups of high or low COL10A1 mRNA levels. The mRNA expression level of COL10A1 was significantly associated with tumor size (*P* = 0.003), lymphatic emboli (*P* = 0.007), lymph node metastasis (*P* = 0.000), serosal invasion (*P* = 0.004), AJCC stage (*P* = 0.000), and recurrence risk (*P* = 0.001) (Table 2). AJCC stage (HR: 2.454, 95% CI: 1.146-4.247; *P* = 0.023) and COL10A1 expression (HR: 2.231, 95% CI: 1.025-5.652; *P* = 0.024) were identified to be independent prognostic covariates for poor overall survival (Supplementary Table 7). Kaplan–Meier curves of overall survival (OS) and recurrence-free survival showed that patients with an elevated COL10A1 mRNA level had a significantly negative prognostic impact with shorter overall and recurrence-free survival (Fig. 2a, b). Subgroup analysis of OS in EGC and AGC groups showed the similar results (Supplementary Fig. 1c, d).

**Table 2:** Correlations between COL10A1 mRNA levels and clinical-pathological parameters of gastric cancer patients

Variable	Patient no.	COL10A1 mRNA		*P*-value
		Low no.(%)	High no.(%)	
Gender				
Male	72	37(51.4)	35(48.6)	0.562
Female	31	14(45.2)	17(54.8)	
Age(years)				
<60	63	36(57.1)	27(42.9)	0.052
≥60	40	15(37.5)	25(62.5)	
Tumor size				
<5 cm	62	38(61.3)	24(38.7)	0.003
≥5 cm	41	13(31.7)	28(68.3)	
Differentiation				
Well	31	21(67.7)	10(32.3)	0.052
Moderate	40	17(42.5)	23(57.5)	
Poor	32	13(40.6)	19(59.4)	
Lymphatic emboli				
No	59	36(61.0)	23(39.0)	0.007
Yes	44	15(34.1)	29(65.9)	
Lymph node metastasis				
No	43	32(74.4)	11(25.6)	0.000
Yes	60	19(31.7)	41(68.3)	
Serosel invasion				
No	44	29(65.9)	15(34.1)	0.004
Yes	59	22(37.3)	37(62.7)	
AJCC stage				
Early	48	33(68.8)	15(31.2)	0.000
Advanced	55	18(32.7)	37(67.3)	
Recurrence				
No	58	37(63.8)	21(36.2)	0.001
Yes	45	14(31.1)	31(68.9)	

Early stage included stage I (*n* = 26) and stage II (*n* = 22). Advanced stage included stage III (*n* = 41) and stage IV (*n* = 14)

### SOX9 and COL10A1 are both up-regulated in GC

The protein levels of SOX9 and COL10A1 in six GC cell lines, including MKN28, MKN45, MGC803, BGC823, SGC7901, and AGS, were measured by western blot analysis. The expression pattern of COL10A1 in these six cell lines was very similar to that of SOX9, excluding MKN28 cell line (Fig. 2c). This finding was similar to the QPCR results obtained for these six lines (Supplementary Fig. 2b). Next, the cellular localization of the two proteins was observed by a two-color immunofluorescence assay. The results demonstrated that the endogenous proteins of SOX9 and COL10A1 were both localized in the cell nucleus and cytoplasm of SGC7901 and AGS. Merged signals of SOX9 and COL10A1 indicated that these two proteins were co-localized (Fig. 2d).

To further investigate the correlation between SOX9 and COL10A1 expression in vivo, we performed QPCR and western blot analysis using 30 pairs of tumor tissues (T) and adjacent normal gastric mucosa (N). The results, as shown in Fig. 2e, indicated that the mRNA expression of SOX9 and COL10A1 were significantly increased in the tumor tissues. The protein expressions levels of SOX9 and COL10A1 were significantly up-regulated both in Stage I and Stage IV groups (Fig. 2g and Supplementary Fig. 1c). Semi-quantitative scoring indicated that both SOX9 and COL10A1 protein levels were significantly higher in tumor tissues (Fig. 2h). After the regression coefficients between the expressions of SOX9 and COL10A1 were calculated, we observed significant correlations between SOX9 and COL10A1 in mRNA and protein levels in primary GC (*R* = 0.7372 and *R* = 0.5768) (Figs. 2f, i).

### SOX9 directly binds to the COL10A1 promoter and activates its transcription

**Fig. 3:**
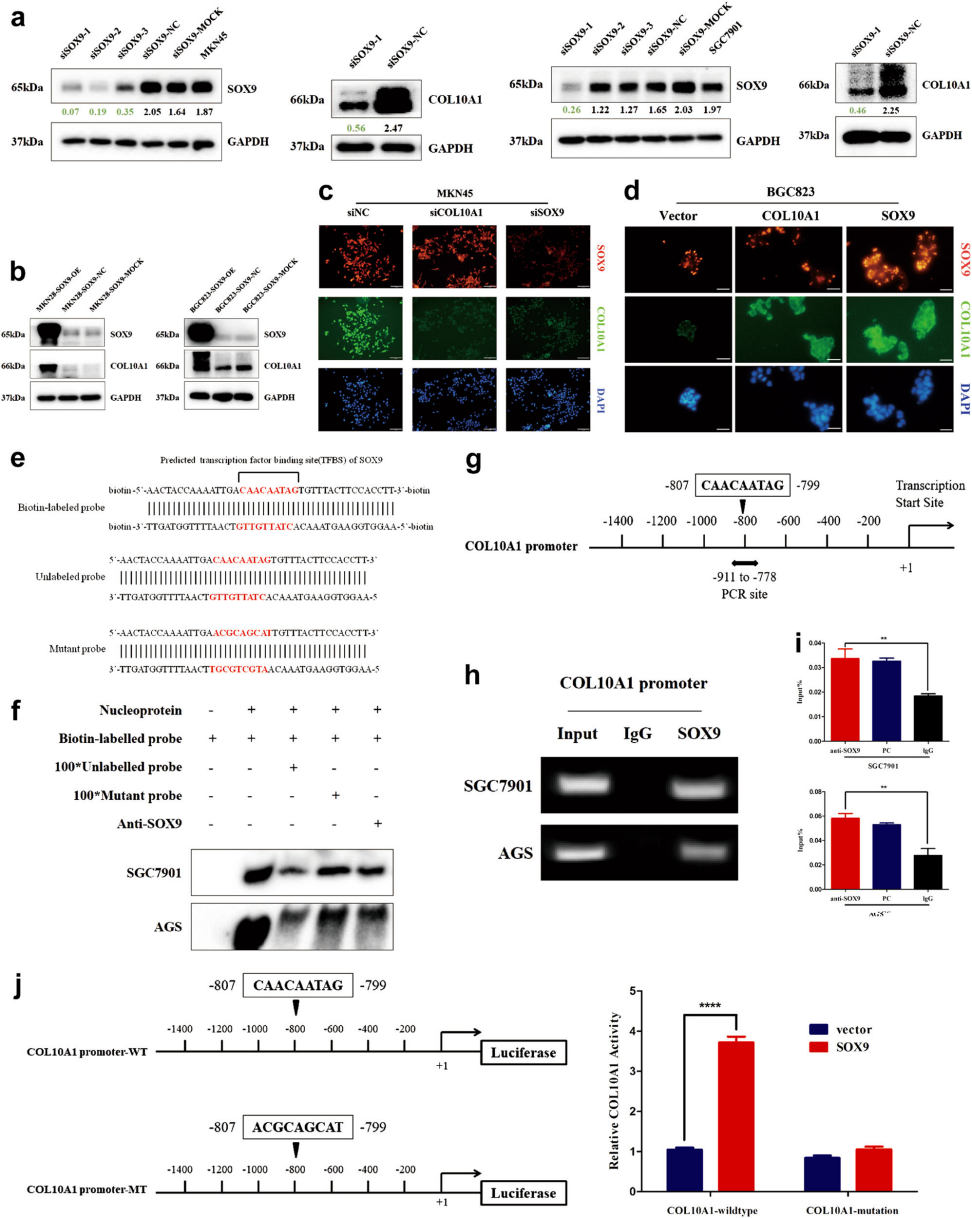
SOX9 up-regulates the protein expression of COL10A1 and its transcriptional activity by directly binding to the COL10A1 promoter. **a**, **b** SOX9 and COL10A1 expression were detected using western blot analysis by transfecting GC cell lines with SOX9 siRNA and SOX9-sense plasmids. **c**, **d** Visualization of SOX9 (red) and COL10A1 (green) staining in SOX9-silenced and COL10A1-silenced MKN45 cells and in SOX9-overexpressing and COL10A1-overexpressing BGC823 cells by immunofluorescence microscopy. **e** The transcription factor-binding site (TFBS) of SOX9 in the COL10A1 gene promoter was used to design biotin-labeled, unlabeled, and mutant probes for EMSA. **f** EMSA using nuclear extracts from SGC7901 and AGS cells is shown. Shifted bands representing that endogenous SOX9 bound to the biotin-labeled probe were detected (lane 2). When a 100-fold excess of unlabeled probe was added to bind to endogenous SOX9 and compete with the biotin-labeled probe, the bands displayed a lower intensity (lane 3). However, when a 100-fold excess of mutant probe that could not bind to endogenous SOX9 was added, the intensity of the bands recovered (lane 4). With the addition of SOX9 antibody, a significantly lower intensity of the band was evident, which further indicated that the SOX9 protein could bind to the TFBS in the biotin-labeled probe (lane 5). **g** Schematic diagram depicting the positions of the primers used for the ChIP assay. **h**, **i** ChIP analysis was performed using a negative control immunoglobulin G (IgG) or anti-SOX9 antibody in SGC7901 and AGS cells. *PC* positive control (anti-RNA polymerase II antibodies). **P < 0.01. **j** Schematic diagram depicting the sequences in the wild type and mutant COL10A1 promoter. *WT* wild type, *MT* mutant type. MKN28 cells were harvested for analysis of luciferase activity and SOX9 protein stimulated COL10A1 activity. Scale bars represent 60 μm in (**c**, **d**)

To further identify whether SOX9 regulated the expression of COL10A1, MKN45 and SGC7901 cell lines were transfected with SOX9 siRNA (Supplementary Table 8). The protein expression of COL10A1 was down-regulated after transfection of SOX9 siRNA in both gastric cell lines (Fig. 3a). When MKN28 and BGC823 were treated with SOX9-sense plasmids, the expression of COL10A1 was induced (Fig. 3b). A weakened intensity of COL10A1 was observed in SOX9-silenced MKN45 cells, while the intensity of SOX9 was not weakened in COL10A1-silenced MKN45 cells (Fig. 3c). The enhanced intensity of COL10A1 was detected in SOX9-overexpressing BGC823 cells, while the intensity of SOX9 was not enhanced in COL10A1-overexpressing BGC823 cells (Fig. 3d).

To identify whether SOX9 directly bound to the COL10A1 promoter in vitro, electrophoretic mobility shift assays (EMSA) were performed. According to the predicted transcription factor-binding site, biotin-labeled, unlabeled and mutant probes were designed and synthesized (Fig. 3e). EMSA experiment showed that SOX9 directly bound to the COL10A1 promoter (Fig. 3f). To identify that SOX9 could directly bind to the COL10A1 promoter in vivo, chromatin immunoprecipitation (ChIP) assay was performed. The putative SOX9-binding site (−807 to −799 bp) (Fig. 3g) exhibited a significant enrichment after immunoprecipitation with an anti-SOX9 antibody. No band was evident after immunoprecipitation with negative control IgG antibody (Fig. 3h). The QPCR results indicated that the protein/COL10A1 gene complexes were pulled down by the anti-SOX9 antibody in SGC7901 and AGS cell lines (Fig. 3i). To confirm the functional link between the SOX9-binding site and COL10A1 promoter activity, wild type (COL10A1-promoter-WT) and mutant type (COL10A1-promoter-MT) reporter gene sequences of the COL10A1 promoter were designed and synthesized (Fig. 3j, left). Overexpression of SOX9 resulted in increased expression of the COL10A1-promoter-WT reporter gene, but it did not increase the expression of the COL10A1-promoter-MT reporter gene (Fig. 3j, right).

### COL10A1 facilitates the malignant cellular-biological behavior of GC

**Fig. 4:**
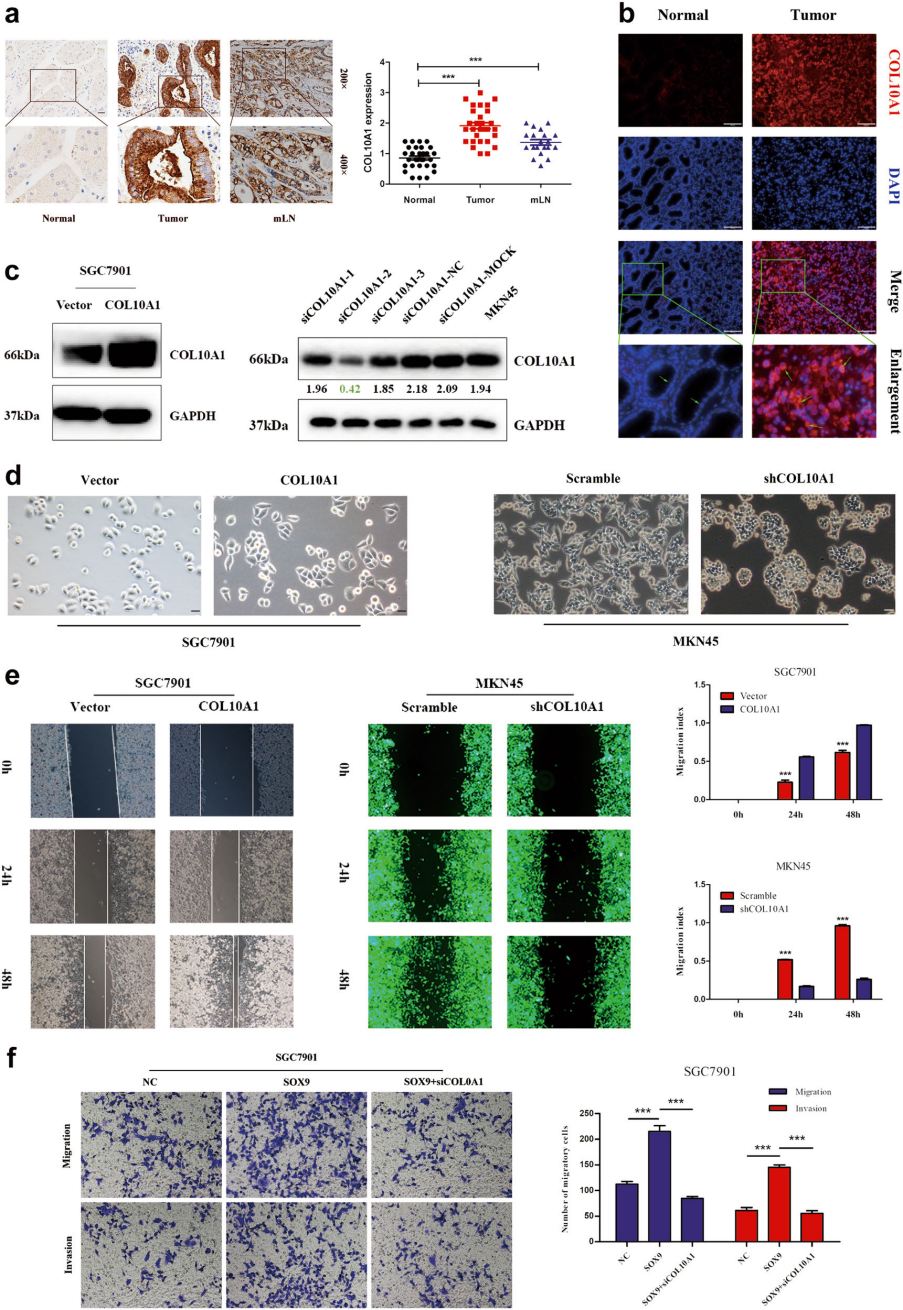
COL10A1 facilitates the malignant biological behavior of gastric cancer. **a** The protein expression of COL10A1 in tumors with mLNs was determined using IHC staining. *mLNs* metastatic lymph nodes. **b** Visualization of COL10A1 (red) staining in normal and GC tissues by immunofluorescence microscopy. **c** COL10A1 protein expression was determined by western blot analysis after transfection of GC cell lines with COL10A1-sense plasmids and COL10A1-siRNA. **d** Phase-contrast microscopy visualization of the morphology of SGC7901 cells by transfection of vector and COL10A1 and MKN45 cells by transfection of COL10A1 siRNA lentivirus. **e** COL10A1 overexpression significantly enhanced the migration ability of cells compared with vector, while knockdown of COL10A1 achieved the opposite effect. ***P < 0.001. **f** Representative figures of transwell assays in SGC7901 and MKN45 cells after treatment with small interfering RNA and/or plasmid are shown. ***P < 0.001. All results shown were reproduced in at least three independent experiments. Scale bars represent 100 μm in (**a**, **b**) and 20 μm in (**d**)

We first detected the protein expression of COL10A1 in GC tissues, matched non-cancerous gastric mucosa and metastatic lymph nodes of 30 patients by immunohistochemistry (IHC) analysis. COL10A1-positive cells could be observed at a high level in the cancer tissues and metastatic lymph nodes (Fig. 4a). As shown in Fig. 4b, the gastric cancer tissues expressed higher levels of COL10A1 compared with the corresponding non-cancerous region using fluoroscopy and immunohistochemistry methods.

Moreover, we established SGC7901 stable transfectants via the transfection of COL10A1-sense plasmids and vector. The functional effect of COL10A1 knockdown on MKN45 cells in vitro was achieved by transfecting COL10A1 siRNA (Supplementary Table 8) lentivirus. The mRNA and protein expression were confirmed by QPCR and western blot analysis (Supplementary Fig. 2d and Fig. 4c). COL10A1 overexpression in the SGC7901 cells displayed the spindle-shaped and fibroblastic-like phenotype. In contrast, stable transfectants of the vector displayed a round, flat or mixed morphology with a short cytoplasmic extension, while knockdown of COL10A1 MKN45 cells achieved the opposite effect (Fig. 4d). The migration ability of COL10A1-overexpression SGC7901 cells was significantly higher than that of vector cells, while knockdown COL10A1 MKN45 cells exhibited the opposite effect based on a wound-healing assay (Fig. 4e).

**Fig. 5:**
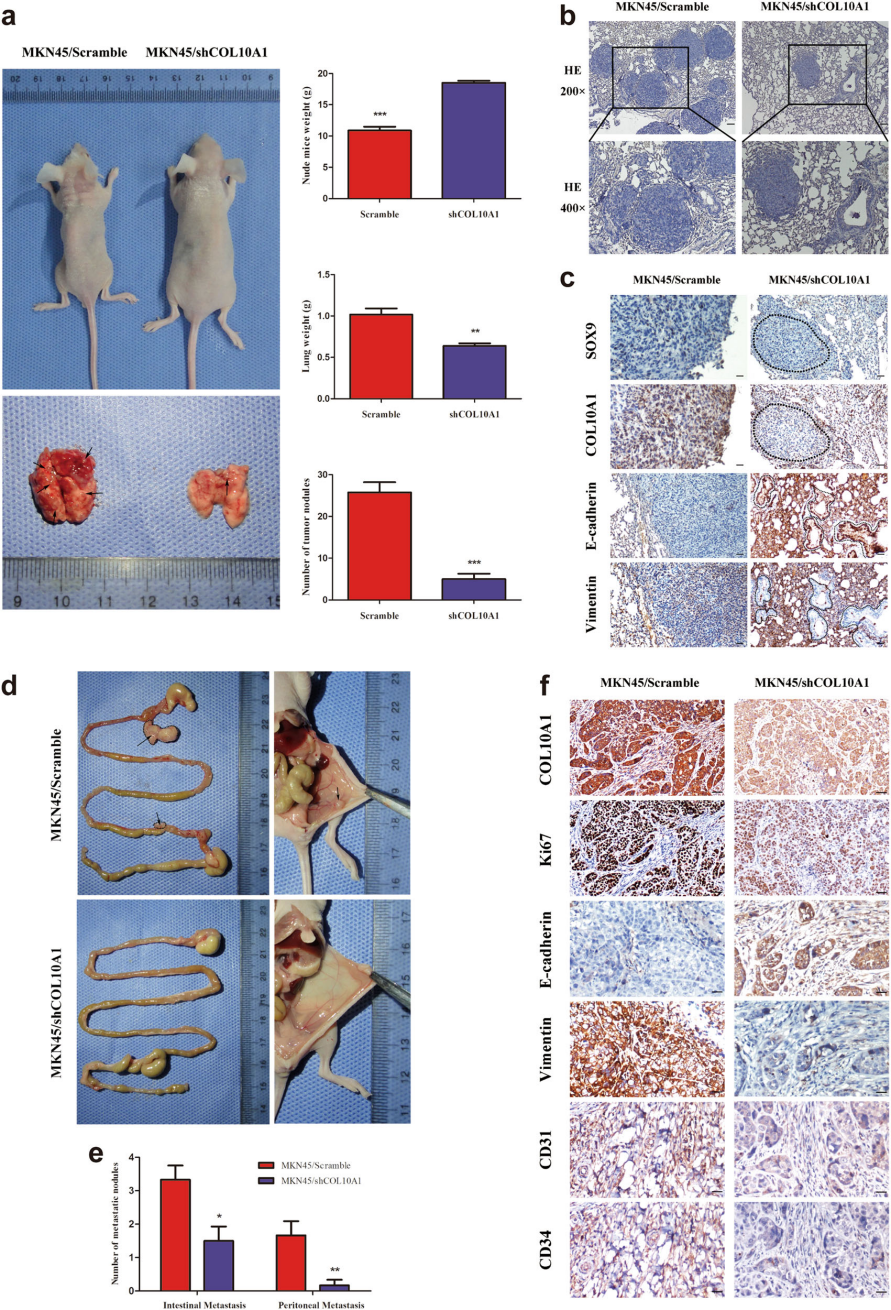
COL10A1 facilitates EMT, lung metastasis, and intestinal and peritoneal metastasis in GC in vivo. **a** Nude mice were injected with tumor cells through the tail vein (*n* = 10 in each group). The weights of the mice, lung weight and number of metastatic nodules in the lung were counted (right). **b** Representative figures of metastatic nodules in the lungs were observed under a microscope. **c** Paraffin-embedded lung metastasis nodules were stained with anti-SOX9, anti-COL10A1, anti-E-cadherin or anti-Vimentin antibodies using IHC. **d** Nude mice were injected with tumor cells through the abdominal cavity to evaluate the intestinal and peritoneal metastatic potential of the cells (*n* = 6 in each group). **e** The intestinal metastasis and peritoneal metastasis per group were counted. **f** Paraffin-embedded peritoneal metastasis sections were stained with anti- COL10A1, anti-Ki67, anti-E-cadherin, anti-Vimentin, anti-CD31 or anti-CD34 antibodies using IHC. Scale bars represent 100 μm in (**a**), 60 μm in (**c**) and 20 μm in (**f**)

We achieved the same results in metastasis assays in vivo by injecting knockdown COL10A1 MKN45 cells into nude mice via the tail vein and abdominal cavity. At 6 weeks, LV-Scramble mice showed significantly greater weight loss and higher lung tissue weights than LV-shCOL10A1 mice. Accordingly, the lung metastatic nodules in the LV-Scramble group were larger than those in the LV-shCOL10A1 group (Figs. 5a, b). The IHC assay of lung metastatic tumors revealed that knockdown of COL10A1 enhanced the expression of E-cadherin (epithelial marker) and decreased the expression of Vimentin (mesenchymal marker), while there was not significant decrease in SOX9 expression (Fig. 5c). Knockdown of COL10A1 MKN45 cells showed weaker metastatic ability and the formation of fewer tumors (including intestinal and peritoneal metastatic tumors) in the abdominal cavities of nude mice (Figs. 5d, e). The IHC assay of E-cadherin and Vimentin revealed that the protein expressions of peritoneal metastatic tumors were consistent with those in lung metastatic tumors (Fig. 5f). Next, we detected the protein expressions of cell proliferation (Ki-67) and angiogenesis (CD31 and CD34) markers in the peritoneal metastatic tumors. Knockdown of COL10A1 showed a significantly decreased growth rate and tumor vessel density compared with scramble group (Fig. 5f).

### SOX9 is essential for COL10A1-mediated EMT and cell migration and invasion in GC

**Fig. 6:**
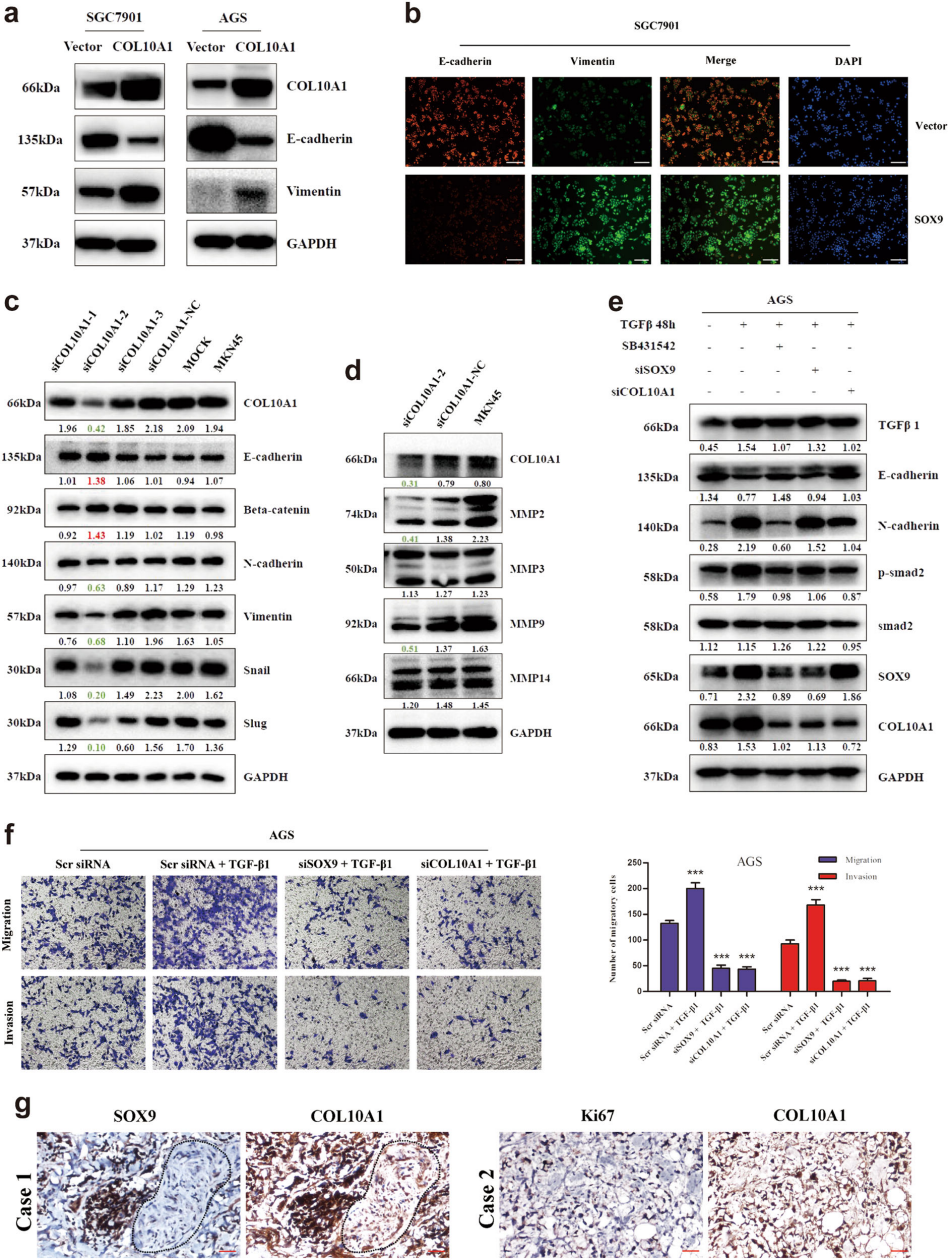
SOX9 is required for COL10A1-mediated EMT and the aggressive cell phenotype. **a**, **b** EMT biomarkers (E-cadherin and Vimentin) were detected after transfection of COL10A1-sense plasmids into the indicated cells using western blot analysis and immunofluorescence. **c** EMT biomarkers were detected after transfection of MKN45 cells with COL10A1-siRNA using western blot analysis. **d** MMPs (MMP 2, 3, 9, and 14) were detected after transfection of MKN45 cells with COL10A1-siRNA using western blot analysis. **e** Proteins extracted from AGS cells co-treated with TGF-β1 and the inhibitor SB431542, SOX9 siRNA or COL10A1 siRNA were used to detect EMT markers, phosphorylated Smad2, SOX9 and COL10A1 using western blotting analysis. **f** Representative figures of transwell assays in AGS cells after treatment with small interfering RNA and/or TGF-β1 are shown. ***P < 0.001. **g** SOX9, COL10A1, and Ki67 expression levels in peritoneal metastatic specimens were detected using IHC. All results shown were reproduced in at least three independent experiments. Scale bars represent 60 μm in (**b**, **g**)

To investigate whether SOX9 regulated the COL10A1-mediated cell aggressive phenotype, we performed a rescue experiment. The results indicated that knockdown of COL10A1 weakened cell migration and invasion in SOX9-overexpressing SGC7901 cells (Fig. 4f). After stable expression of COL10A1 in SGC7901 and AGS cells, an up-regulation of Vimentin (a mesenchymal marker) and down-regulation of E-cadherin (an epithelial marker) were confirmed (Fig. 6a). The same result was observed in the immunofluorescence analysis of SGC7901/COL10A1 cells (Fig. 6b). Up-regulation of E-cadherin and Beta-catenin (epithelial markers) and downregulation of N-cadherin, Vimentin, Snail and Slug (mesenchymal markers) were observed by knockdown of COL10A1 in MKN45 cells (Fig. 6c). The protein expression levels of BMP4 and TGF-β1 were downregulated after COL10A1 knockdown (Supplementary Fig. 3d). Moreover, MMP-2 and MMP-9 (mesenchymal markers) were downregulated, while MMP3 and MMP14 were unaltered (Fig. 6d).

To elucidate the relationship between SOX9 and the TGF-β signaling pathway, we analyzed the effect of recombinant TGF-β1 in GC cells. The data demonstrated that TGF-β1 stimulation caused a significant change in classical EMT markers, such as E-cadherin, N-cadherin, Vimentin, Snail and Slug. Moreover, we observed an increase in the expression of COL10A1 and SOX9 in response to TGF-β1 stimulation in a dose- and time-dependent manner (Supplementary Fig. 3a and b). TGF-β1 treatment at a dose of 2 ng/ml activated Smad2, a key mediator of TGF-β signaling pathway through Smad2 phosphorylation. The phosphorylation levels of Smad2 were upregulated after TGF-β1 stimulation, and the total amount of Smad2 protein was unaltered (Supplementary Fig. 3c). As expected, the TGF-β1 receptor inhibitor SB431542, COL10A1 siRNA or SOX9 siRNA could suppress Smad2 phosphorylation and counteract EMT induced by TGF-β1 stimulation, respectively (Fig. 6e). TGF-β1 stimulation induced a significantly higher capacity for migration and invasion in GC cells compared with Scramble siRNA cells. The capacity for migration and invasion of TGF-β1-induced cells was significantly down-regulated in SOX9 siRNA-transfected and COL10A1 siRNA-transfected cells (Fig. 6f).

Furthermore, we detected the protein expression of SOX9, COL10A1, Ki-67 (cell proliferation marker), CD31 and CD34 (angiogenesis markers) in peritoneal metastatic tumors of GC patients. Representative IHC staining images are shown in Fig. 6g (SOX9, COL10A1 and Ki-67) and Supplementary Figure 3e (CD31 and CD34). The expression of SOX9 was consistent with the expression of COL10A1 in peritoneal metastatic tumors. The clinical samples exhibited a high growth rate and vessel density in peritoneal metastatic tumor tissues.

### Co-expression of SOX9 and COL10A1 correlates with a poor prognosis in human GC

**Fig. 7:**
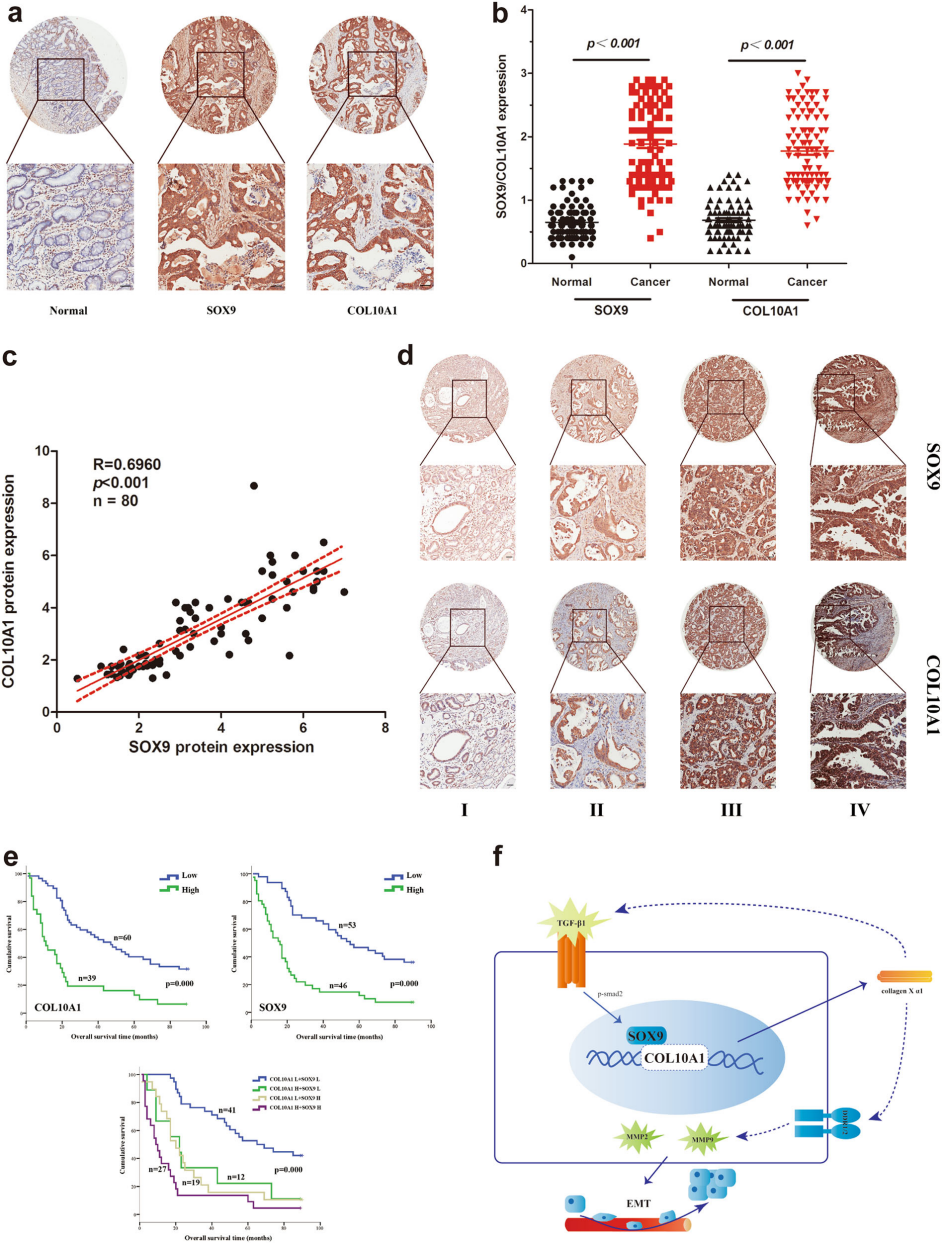
Co-expression of SOX9 and COL10A1 correlates with a poor prognosis in human GC. **a** IHC staining of SOX9 and COL10A1 was performed in the TMA containing 99 gastric cancer patients. **b** SOX9 and COL10A1 protein expression levels in normal and GC tissues. ***P < 0.001 between normal and GC tissues. **c** The protein expression levels of SOX9 and COL10A1 were semi-quantified, and the correlation between these two proteins was evaluated with Spearman’s rank correlation method. ***P < 0.001. **d** IHC staining of SOX9 and COL10A1 expression in human GC (AJCC stages I–IV). **e** Overall survival of GC patients was analyzed using the Kaplan-Meier method. Survival analysis was performed according to the expression of SOX9 and COL10A1. Co-expression of SOX9 and COL10A1 was associated with a poor prognosis in GC patients. **f** A hypothetical model demonstrating that the TGF-β1-SOX9 axis contributes to the transcriptional activation of COL10A1 and promotes GC aggressiveness. Scale bars represent 100 μm in (**a**, **d**)

We performed IHC staining using a tissue microarray (TMA) to identify the clinical relevance of SOX9 and COL10A1 in GC. The protein expression levels of SOX9 and COL10A1 were significantly upregulated in GC tissues and showed high concordance (Fig. 7a). The protein expression levels of COL10A1 and SOX9 were significantly correlated with tumor size (*P* = 0.016 and *P* = 0.023), tumor differentiation (*P* = 0.005 and *P* = 0.022), lymph node metastasis (*P* = 0.004 and *P* = 0.001), and serosal invasion (*P* = 0.001 and *P* = 0.015). The protein expression level of COL10A1 and SOX9 were associated with AJCC stage (*P* = 0.004 and *P* = 0.001) by Pearson’s chi-square tests (Supplementary Table 9). Representative images of the tumors of different stages after IHC staining were shown in Fig. 7d. AJCC stage (HR: 2.023, 95% CI: 1.046-3.912; *P* = 0.036), COL10A1 expression (HR: 2.586, 95% CI: 1.336-4.022; *P* = 0.024) and SOX9 expression (HR: 1.567, 95% CI: 1.483-3.093; *P* = 0.031) were identified to be independent prognostic covariates for poor overall survival in multivariate analysis (Supplementary Table 10).

Semiquantitative scoring showed that the protein expression levels of SOX9 and COL10A1 in GC tissues were significantly higher (Fig. 7b). We observed a significant linear correlation between SOX9 and COL10A1 (*R* = 0.6960, *P* < 0.001) in primary GC (Fig. 7c). The Kaplan–Meier survival curves indicated that high expression of SOX9 or COL10A1 was associated with a poor survival. The double-positive cases (expressing both proteins) displayed the worst disease prognosis (Fig. 7e).

## Discussion

The principal aims of this study were to investigate the role of COL10A1 in cell invasiveness and metastasis and to assess the clinical prognostic value of COL10A1 mRNA and protein expression in GC patients. The principal finding was that COL10A1 was overexpressed in GC tissues and predicted a poor clinical outcome of GC patients for the first time. We further identified a role for COL10A1 in promoting EMT. Moreover, our results indicated that COL10A1 might promote tumor aggressiveness via upregulation of the TGF-β1-SOX9 axis. In addition, our results revealed the SOX9-binding site in the functional region of the COL10A1 promoter and found that SOX9 directly regulated the gene expression of COL10A1. SOX9 was identified as a critical determinant in COL10A1-mediated EMT and metastasis.

Collagen type X alpha 1 (COL10A1) is a member of the collagen family and has not been reported in GC. Previous evidence has indicated that a collagen-rich microenvironment plays an important role in tissue architecture and serves as a barrier to the migration of epithelial cells under normal conditions. However, abnormal ECM can promote abnormal behavior in cancer cells and tumor progression^27,28^. Previous studies have indicated that enhanced collagen cross-linking and deposition are associated with an increase in breast cancer risk^29,30^. Furthermore, increased collagen deposition and cross-linking increase interstitial pressure. Tumor cells are sensitive to changes in the stiffness of their environment, specifically increased collagen crosslinking, which then drives them toward the malignant phenotype^27,28^.

The TGF-β/BMP/SMAD signaling pathway has been studied for a long time and has pivotal roles in cancer progression and metastasis^31–33^. The membrane receptor, serine/threonine kinase complex, can be activated by TGF-β1 and phosphorylate the transcription factors Smad1, Smad2 or Smad3 and then bind to Smad4, and travel to the nucleus^34–36^. Yamamura S et al^37^. characterized that TGF-β signaling pathway was activated in peritoneal metastases and an inhibitor of A-83-01, which has been reported to inhibit Smad signaling, could improve cancer survival. Our findings suggested that the expression of SOX9, COL10A1 and p-Smad2 was enhanced by TGF-β1 stimulation in GC cells, further indicating that COL10A1 might play an important role in the TGF-β1/Smad2 downstream signaling pathway.

Epithelial-mesenchymal transition (EMT) is a differentiation process of the epithelial cell phenotype into the mesenchymal cell phenotype, which includes an enhanced migratory and invasive capacity, increased tumor resistance to apoptosis, and greatly elevated ECM production^7,8,38^. The importance of the TGF-β signaling pathway in EMT has been clearly studied, and TGF-β1 can induce EMT via the Smad2/3-dependent pathway in various epithelial cells and transgenic mice^39,40^. In our study, TGF-β1 stimulation induced decreased E-cadherin expression, increased Vimentin, Snail and Slug expression, and promoted the migratory and invasive capacity of GC cells. In contrast, COL10A1 knockdown slowed TGF-β1-induced EMT. The results indicated that COL10A1 might act as a potent co-stimulator of TGF-β1-induced EMT in GC.

This study also demonstrated that the potential binding site of the COL10A1 promoter (CAACAATAG) was necessary for activation of transcription by SOX9. EMSA, quantitative ChIP assays and a dual-luciferase reporter assay further indicated that the potential binding site played a critical role in the upregulation of COL10A1 transcription. Our results showed that SOX9 could increase the transcription of COL10A1 by directly binding to the potential binding site. Moreover, TGF-β1 stimulation resulted in increased SOX9 expression, which could directly bind to the COL10A1 promoter and activate its transcription. Our findings suggest that TGF-β1 may upregulate COL10A1 expression through the transcription factor SOX9.

COL10A1 knockdown was associated with decreased matrix metalloproteinase (MMP2 and MMP9) protein expression (Fig. 6d). MMP2 and MMP9 have been shown to be upregulated in angiogenic lesions, and angiogenesis is required for the growth and metastasis of invasive tumors and plays an important role in the control of cancer progression^41,42^. Thus, MMP2 and MMP9 appear to have a vital role in GC progression associated with the overexpression of COL10A1. In our study, we further found that the high protein expression of CD31 and CD34 (angiogenesis markers) was consistent with the high protein expression of COL10A1 in the peritoneal metastatic tumors of GC patients. The mechanism whereby COL10A1 mediates these effects is currently unknown. Previous research has shown that the discoidin domain receptor (DDR) family, including DDR1/2, is associated with collagen-mediated EMT and metastatic phenotypes^43,44^. Gastric cancer cells express DDR1/2, and DDR1/2 are associated with the development of GC, based on a search of the GeneCards database, Oncomine (Supplementary Fig. 4) and some studies^45–47^. However, for COL10A1 protein, very little is known about its relationship with DDR or other downstream signaling pathways (Fig. 7f). Overall, further research to explore the mechanisms of the downstream signaling pathway regulated by COL10A1 is needed, and approaches to modulate the transcription or translation of COL10A1 would be therapeutically beneficial.

## Conclusion

Our mRNA expression profiling analyses have indicated that COL10A1 is significantly elevated during the development and progression of GC. The expression of COL10A1 is markedly overexpressed in GC patients and enhances malignant and metastatic ability in vivo. Increased mRNA and protein expression of COL10A1 are associated with shorter survival in GC patients. Moreover, COL10A1 promotes cell migration and invasion through positive transcriptional regulation of SOX9 and the involvement of the TGF-β signaling pathway. Therefore, this study not only elucidates the precise mechanism of COL10A1 in the progression of GC but also provides molecular insight into cancer-related ECM and may serve as a predictor of clinical outcome in GC.

## Materials and methods

### Patients and tumor tissue specimens

We obtained human GC specimens and corresponding normal gastric tissues of 30 patients who were diagnosed with GC that was confirmed by histopathological examination and had undergone surgical resection in the Department of General Surgery in Nanfang Hospital. Patients’ staging data were classified according to AJCC guidelines. The Ethics Committee of Nanfang Hospital approved the study and we obtained each patient’s written informed consent. Six tumors and six noncancerous gastric tissues that met the criteria for RNAseq (sufficient amount and quality of RNA) were included in this study. The quality of the RNA of the samples profiled for RNAseq analyses are summarized in Supplementary Figure 1a and b.

### RNA-seq library preparation and Hiseq2500 sequencing

TRIzol Reagent (Takara) was used to extract total RNA according to the manufacturer’s protocol. Quant-ITTM RiboGreen ® RNA Reagent (Invitrogen, Carlsbad, CA, USA) was used to measure total RNA concentrations. The Agilent RNA 6000 Nano kit (Agilent 2100 Bioanalyzer) was used to assess the integrity of total RNA. Libraries for Illumina sequencing were constructed from 10 µg of RNA using the NEBNext mRNA Sample Prep Kit 1 (New England Biolabs, Ipswich, MA, USA) according to the manufacturers’ guidelines. The cDNA libraries were purified, and the relative concentrations were measured using the QIAquick PCR kit (Qiagen, Tokyo, Japan). Twelve libraries from cancer and normal tissues were analyzed on one lane of an Illumina Hiseq2500 platform, generating 125-bp paired-end reads. The cleaned sequencing reads were then mapped to the human reference genome (build hg. 19) with TopHat v1.0.12^48^.

### Transcript abundance estimation

Sequencing results were analyzed using Cufflinks software^49^ to calculate the transcription abundance for each gene. The expression level of each transcript was calculated based on values for fragments per kilobase of exon model per million fragments mapped (FPKM). With Cuffdiff, RNA-seq can now be used to determine differentially expressed genes. Following the Benjamini-Hochberg procedure for multiple tests^50^, the criteria for significant differences was selected using the false discovery rate (F.D.R) < 0.05.

### Tissue microarray (TMA)

A constructed TMA containing 99 gastric cancer cases was obtained from the National Engineering Center for Biochip (Shanghai, China). Information for postsurgical pathological staging and overall survival time for all cases (Supplementary Table 1) was available to analyze the clinical relevance.

### Immunohistochemistry (IHC)

Fresh 5-μm sections were cut from paraffin-embedded gastric tissue samples. After the sections were baked (65 °C, 1 h), they were deparaffinized in xylene, followed by re-hydration in graded ethanol solutions. The sections were boiled with citrate buffer for 5 min for antigenic retrieval, followed by incubation with 1% BSA as a blocking agent for prevention of nonspecific binding. The sections were incubated overnight at 4 °C. As a negative control, normal IgG was used to verify the antibody specificity. After washing, anti-rabbit or anti-mouse secondary antibody (Zhongshan Biotech, Beijing, China) was used. The sections were incubated with DAB (3,3-diaminobenzidine), counterstained, dehydrated and mounted in permanent mounting medium. Two pathologists independently evaluated and scored the sections, and the IHC score was estimated as the average score. The sections were scored for the intensity of staining based on a scale of 0 (no staining), 1 (weak staining, light yellow), 2 (moderate staining, yellowish brown), and 3 (strong staining, brown). Negative and weakly stained cells were considered low, while moderately and intensely stained cells were defined as high expressers of this protein.

### GC cell lines and cell culture

The GC cell lines MKN28, MKN45, MGC803, BGC823, SGC7901 and AGS were obtained from the American Type Culture Collection (ATCC, Manassas, VA, USA). The authentication of human GC cell lines was achieved by short tandem repeat (STR) profiling, and cell lines were maintained in culture less than 3 months after authentication. The GC cells were cultured in RPMI 1640 medium (Invitrogen) containing 10% FBS (Gibco) at 37 °C, 5% CO_2_. The GC cells were stimulated with human recombinant TGF-β1 diluted with serum-free medium (0.5, 1, 2, 5 or 10 ng/ml; Peprotech, London, UK), for 24 and 48 h. The TGF-βRI/ALK5 inhibitor SB431542 (Selleck, Shanghai, China) was added to the medium at the appropriate time.

### Quantitation of mRNA by real-time PCR

After harvesting the cells, total RNA was purified using TRIzol Reagent (Takara). Then, mRNA was reverse-transcribed into cDNA using a PrimeScript^TM^ RT reagent kit (Takara). The primers synthesized by Takara are listed in Supplementary Table 2. We performed real-time PCR to quantitate mRNA expression as described in the protocol supplied with SYBR Premix Ex Taq (Takara) using a LightCycler 480 v.1.5 system (Roche). SOX9 and COL10A1 gene expression were normalized to GAPDH gene expression (internal control) using the 2-ΔΔCt method^51,52^.

### Western blot analysis

The proteins were dissociated and separated by SDS/PAGE, and then transferred to PVDF membranes, which were incubated with primary antibodies. The following primary antibodies were used: rabbit anti-SOX9, anti-MMP2, anti-MMP3, anti-MMP9, anti-MMP14, anti-BMP4, anti-TGFβ1, anti-Smad2, anti-Smad3, anti-p-Smad2 (phosphor S255), anti-p-Smad3 (phosphor S423 + S425) and mouse anti-COL10A1 (Abcam, Cambridge, MA, USA); rabbit anti-Beta-catenin, anti-E-cadherin, anti-N-cadherin, anti-Vimentin, anti-Snail, anti-Slug, and anti- GAPDH (Cell Signaling Technology, Danvers, MA, USA). Antigen-antibody complexes were detected using horseradish peroxidase-conjugated secondary antibodies (Cell Signaling Technology) with ECL western blot detection reagent (Merck Millipore). Protein expression was quantified using US National Institutes of Health (NIH) ImageJ software.

### SiRNA transfection and lentiviral infection

Specific or scrambled short interfering RNAs (siRNAs, GenePharma, Shanghai, China) were used to transfect GC cells using Lipofectamine 3000 (Invitrogen). The siRNA sequences targeting SOX9 and COL10A1 are shown in Supplementary Table 8. The most efficient COL10A1 sequence was selected to generate a stable cell line (LV-COL10A1) via lentiviral delivery of short hairpin RNAs (GeneChem, Shanghai, China). According to the manufacturer’s instructions, MKN45 cells were infected with LV-Scramble or LV-COL10A1. The infection efficiency can be evaluated by analyzing GFP-infected and puromycin-selected cells within 2–3 weeks with a flow cytometer.

### Establishment of stable transfectants

Human full-length SOX9 and COL10A1 complementary DNA (cDNA) was amplified by RT-PCR. The products were subsequently cloned into the expression vector of pEnter/Flag (Vigene Biosciences, Jinan, China). To establish stable cell lines, empty pEnter vector or pEnter-SOX9 or pEnter-COL10A1 were used to transfect GC cells. The transfected cells were passaged at 1:15 and selected in RPMI 1640 medium supplemented with puromycin (2 µg/ml) for 4 weeks. SOX9 and COL10A1 expression were assessed by western blot analysis.

### Immunofluorescence microscopy

Mouse anti-COL10A1 (Abcam), rabbit anti-SOX9 (Abcam), anti-E-cadherin, anti-vimentin primary antibodies (CST) and appropriate fluorophore-conjugated secondary antibodies (KeyGEN, Nanjing, China) were used for immunofluorescence labeling. The nucleus was visualized with 1 μg/mL DAPI. Images were then taken using an Olympus confocal microscope (Olympus Fluoview 500 IX71) or a fluorescence microscope.

### Wound healing assay

A diluted cell suspension was prepared and seeded into a 6-well plate at 3 × 10^5^ cells/well and cultured until 100% confluence. The monolayer was wounded using a 10 µl pipette tip, and then cells were washed to remove cell debris and cultured in RPMI 1640 culture medium with 1% FBS. The wound edges were observed under an inverted microscope at 0, 24, and 48 h and measured using NIH ImageJ software.

### Transwell assay

In vitro migration and invasion assays were examined using Transwell cell culture chambers (8 μm pore size). Upper membranes were coated with Matrigel (BD Biosciences) as a barrier (50 μg/well) for invasion assay. Cells (1 × 10^5^) were placed into the upper chamber. After incubation at 37 °C, 5% CO_2_ for 48 h, the chambers were placed in 4% paraformaldehyde for 30 min and then stained with 2% crystal violet for 1 h. Cells that migrated toward the outer chamber were counted in five representative (200×) fields per insert.

### Electrophoretic mobility shift assay (EMSA)

We performed EMSA to validate the SOX9 and COL10A1 binding activities in the nucleus using a lightshift chemiluminescent EMSA kit (Beyotime, Shanghai, China) according to the manufacturer’s instructions. Biotin-labeled and unlabeled probes as competitors for SOX9-specific binding were designed as follows: 5′ AACTACCAAAATTGACAACAATAGTGTTTACTTCCACCTT -3′ (forward); and 5′-AAGGTGGAAGTAAACACTATTGTTGTCAATTTTGGTAGTT 3′ (reverse).

### Promoter reporter and dual luciferase assays

To elucidate the mechanism by which SOX9 regulates the expression of COL10A1, we performed promoter assays. The full-length human SOX9 cDNA was ligated into the promoter-driven luciferase (Luc) reporter plasmid pGL3. The luciferase reporter plasmids containing wild type or mutant COL10A1 promoter sequences were constructed by GeneChem Co. (Shanghai, China). The reporter plasmids and pEnter-SOX9 vector were co-transfected into the cells using Lipofectamine 3000 solution (Invitrogen). At 48 h after transfection, we performed luciferase assays using a luciferase reporter assay system (Promega, Madison, WI, USA). Luciferase/b-galactosidase activity was used to calculate luciferase activity.

### ChIP assay

The GC cells were treated with 1% formaldehyde for 10 min to cross-link proteins to DNA. Next, we performed a ChIP assay using the ChIP Assay Kit (Beyotime, Shanghai, China) according to the manufacturer’s instructions. Equal aliquots of precleared chromatin were subjected to sonication followed by immunoprecipitation with a specific antibody directed against SOX9, RNA polymerase II and IgG monoclonal antibodies. The following COL10A1-specific primers were used: 5′-GGACTCTCAACTTCCCACCTTT-3′ (forward); and 5′-AAACACTTTCCCTCAAAGGTGGA-3′ (reverse).

### Metastasis animal model

For the in vivo metastasis assays, the tail veins of mice were injected with tumor cells at a density of 5 × 10^6^ in 100 μl serum-free RPMI 1640 (*n* = 10 per group, male BALB/c nu/nu, 4–6 weeks old; Laboratory Animal Unit, Southern Medical University, Guangzhou, China). Body weight was measured every 3 days after caudal vein injection. Mice were sacrificed after 21 days. Lung tissue was dissected, imaged and weighed. The lung metastases were detected by H&E and analyzed by IHC staining. The abdominal cavities of mice were injected with tumor cells at a density of 1 × 10^7^ in 200 μl serum-free RPMI 1640 (*n* = 6 per group, male BALB/c nu/nu, 4–6 weeks old; Laboratory Animal Unit, Southern Medical University, Guangzhou, China). Mice were sacrificed after 28 days. The intestinal and peritoneal metastatic tumor tissues were dissected and imaged. The peritoneal metastases were analyzed by IHC staining.

### Statistical analysis

Statistical analyses were performed using the SPSS 20.0 statistical software package (SPSS Inc, Chicago, Illinois, USA). Quantitative data were presented as the mean ± SD of three independent experiments and analyzed by Pearson’s *χ*^2^ or the Student’s *t* test. The relationship between SOX9 and COL10A1 was revealed by linear regression analysis. A survival curve was estimated using the Kaplan–Meier method and compared by two-sided log-rank tests and Wilcoxon tests. Cox proportional hazards regression was used to determine the univariate and multivariate hazard ratios for selected potential parameters. *P*-values < 0.05 (2-sided) were considered significant.

## Electronic supplementary material

Supplementary Table

Supplementary Figure 1

Supplementary Figure 2

Supplementary Figure 3

Supplementary Figure 4

Supplementary figure legends
