# Supplementary material for: TGF-β1-SOX9 axis-inducible COL10A1 promotes invasion and metastasis in gastric cancer via epithelial-to-mesenchymal transition
Source: Cell Death Dis. 2018 Aug 28;9(9):849. doi: 10.1038/s41419-018-0877-2 (PMC6113209; doi:10.1038/s41419-018-0877-2)
Supplement: Supplementary file 1 — Supplementary Table [file 41419_2018_877_MOESM1_ESM.doc]

**Supplementary Table 1**. The clinical-pathological characteristics of 99 clinical samples in TMA

| Sample ID | Gender | Age  (years) | Differentia-  tion | Tumor-size | AJCC stage | Status | Survival time  (months) |
| --- | --- | --- | --- | --- | --- | --- | --- |
| 1 | Male | 79 | Poor | 4.5 | III | Dead | 21 |
| 3 | Male | 52 | Poor | 7 | III | Dead | 24 |
| 4 | Male | 48 | Poor | 2.5 | IIA | Dead | 43 |
| 5 | Male | 77 | Well | 7 | IV | Dead | 6 |
| 6 | Male | 59 | Well | 7 | IV | Dead | 3 |
| 7 | Male | 77 | Well | 3 | IIB | Dead | 9 |
| 8 | Female | 69 | Moderate | 6 | IIIA | Dead | 2 |
| 9 | Male | 73 | Moderate | 2 | IB | Dead | 9 |
| 10 | Female | 62 | Moderate | 3 | IA | Alive |  |
| 11 | Male | 61 | Well | 11 | III | Dead | 11 |
| 12 | Female | 75 | Poor | 1.8 | II | Dead | 63 |
| 13 | Male | 71 | Well | 7.5 | III | Dead | 1 |
| 14 | Male | 41 | Moderate | 6.5 | IIIC | Dead | 8 |
| 15 | Female | 69 | Poor | 6 | IIA | Alive |  |
| 16 | Female | 67 | Moderate | 2.5 | IIIB | Dead | 74 |
| 17 | Male | 65 | Moderate | 3 | IIA | Alive |  |
| 18 | Male | 60 | Well | 12 | IIIA | Dead | 22 |
| 19 | Female | 62 | Moderate | 18 | IIIA | Dead | 16 |
| 20 | Female | 53 | Well | 8 | IIIC | Dead | 19 |
| 21 | Male | 53 | Well | 3.5 | IIB | Dead | 55 |
| 22 | Female | 73 | Well | 3 | IIIA | Dead | 23 |
| 23 | Female | 51 | Moderate | 5 | IIIA | Dead | 8 |
| 24 | Male | 71 | Moderate | 4.5 | IIB | Dead | 17 |
| 25 | Male | 48 | Moderate | 8.5 | IIIA | Alive |  |
| 26 | Male | 72 | Well | 7 | IIIB | Dead | 17 |
| 27 | Male | 70 | Well | 8 | IB | Alive |  |
| 28 | Female | 77 | Well | 6 | IIIB | Dead | 20 |
| 29 | Male | 75 | Well | 4 | IIA | Dead | 50 |
| 30 | Female | 80 | Well | 3.5 | IA | Alive |  |
| 31 | Male | 72 | Well | 15 | IIIA | Dead | 4 |
| 32 | Male | 79 | Moderate | 10 | IIA | Alive |  |
| 33 | Female | 63 | Well | 3 | IIA | Alive |  |
| 34 | Male | 65 | Moderate | 2 | IIB | Dead | 22 |
| 35 | Female | 68 | Poor | 7 | IIIB | Dead | 12 |
| 36 | Male | 78 | Well | 3.5 | IIIA | Alive |  |
| 37 | Female | 57 | Well | 7 | IV | Dead | 17 |
| 38 | Male | 45 | Well | 4 | IV | Dead | 23 |
| 39 | Female | 50 | Well | 2.5 | IIIA | Dead | 17 |
| 40 | Male | 76 | Moderate | 6 | IIIA | Dead | 9 |
| 41 | Male | 65 | Moderate | 4 | II | Dead | 65 |
| 42 | Male | 71 | Moderate | 3.5 | IV | Dead | 17 |
| 43 | Female | 75 | Well | 3 | II | Dead | 85 |
| 44 | Male | 32 | Poor | 7 | IV | Dead | 3 |
| 45 | Male | 58 |  | 8 | IIA | Alive |  |
| 46 | Female | 73 | Moderate | 14 | IIIA | Dead | 9 |
| 47 | Female | 73 | Moderate | 4 | IIIB | Dead | 20 |
| 48 | Female | 58 | Moderate | 4 | IIB | Alive |  |
| 49 | Male | 63 | Poor | 5 | IIIB | Alive | 90 |
| 50 | Male | 59 | Poor | 3 | IB | Alive | 90 |
| 51 | Female | 54 | Well | 7 | IIA | Dead | 20 |
| 52 | Male | 56 | Well | 6 | IIA | Dead | 73 |
| 53 | Male | 62 | Well | 4.5 | IA | Alive | 90 |
| 54 | Female | 73 | Well | 4 | IIIA | Dead | 15 |
| 55 | Male | 68 | Well | 3.5 | IIIA | Dead | 23 |
| 56 | Male | 52 | Moderate | 15 | IIIB | Dead | 22 |
| 57 | Male | 50 | Moderate | 6 | IIIB | Dead | 28 |
| 58 | Female | 74 | Moderate | 5 | IV | Dead | 7 |
| 59 | Female | 55 | Poor | 4 | IIB | Dead | 25 |
| 60 | Female | 67 | Poor | 10 | IIIC | Dead | 3 |
| 61 | Male | 53 | Poor | 5.5 | IIIA | Alive | 90 |
| 62 | Male | 65 | Well | 1.2 | IIB | Alive | 90 |
| 63 | Female | 52 | Moderate | 5 | IIIB | Dead | 48 |
| 64 | Female | 65 | Poor | 7 | IIIB | Dead | 30 |
| 65 | Male | 47 | Well | 6 | IIIB | Dead | 38 |
| 66 | Male | 72 | Moderate | 4.5 | IIIA | Dead | 69 |
| 67 | Male | 62 | Moderate | 3.5 | II | Alive | 89 |
| 68 | Male | 66 | Moderate | 6 | IIA | Alive | 89 |
| 69 | Female | 67 | Moderate | 3.5 | IIA | Alive | 89 |
| 70 | Female | 74 | Well | 5 | IIIA | Dead | 53 |
| 71 | Male | 77 | Poor | 6 | IIB | Dead | 34 |
| 72 | Female | 57 | Moderate | 9 | IIA | Dead | 60 |
| 73 | Female | 67 | Moderate | 3.5 | II | Dead | 69 |
| 74 | Male | 54 | Moderate | 7 | IIA | Dead | 19 |
| 75 | Male | 62 | Well | 5 | IIIB | Dead | 11 |
| 76 | Female | 81 | Well | 2.8 | IIA | Dead | 43 |
| 77 | Female | 81 | Well | 5 | IIIA | Dead | 3 |
| 78 | Male | 62 | Well | 1.8 | IA | Dead | 40 |
| 79 | Male | 62 | Well | 5.5 | IIIC | Dead | 17 |
| 80 | Female | 77 | Well | 10 | IIA | Dead | 47 |
| 81 | Male | 67 | Moderate | 15 | IIIB | Dead | 12 |
| 82 | Male | 79 | Moderate | 7.5 | IIIC | Dead | 10 |
| 83 | Male | 53 | Well | 6 | IIA | Dead | 20 |
| 84 | Male | 66 | Well | 15 | III | Dead | 57 |
| 85 | Male | 49 | Moderate | 20 | IIIB | Dead | 21 |
| 86 | Male | 73 | Well | 14 | IIIC | Dead | 4 |
| 87 | Male | 70 | Well | 6 | IB | Dead | 4 |
| 88 | Male | 72 | Poor | 5 | IIA | Dead | 36 |
| 89 | Female | 59 | Moderate | 3 | IB | Alive | 89 |
| 90 | Female | 58 | Moderate | 5 | IIIB | Alive | 89 |
| 91 | Female | 57 | Well | 3 | IB | Alive | 89 |
| 92 | Male | 78 | Well |  | IIIB | Alive | 89 |
| 93 | Male | 76 | Poor | 7.5 | IIB | Alive | 89 |
| 94 | Male | 56 | Well |  | IIIA | Alive | 89 |
| 95 | Male | 51 | Well | 7 | IIIA | Alive | 89 |
| 96 | Male | 52 | Well | 3 | IIIA | Alive | 89 |
| 97 | Male | 61 | Well | 5 | IIIA | Alive | 89 |
| 98 | Male | 72 | Moderate | 6 | III | Alive | 89 |
| 99 | Male | 74 | Well | 4 | IIB | Alive | 89 |

**Supplementary Table 2**. Primers used for RT-PCR

| Gene | Primer | | Accession |
| --- | --- | --- | --- |
| *COL10A1* | Forward | 5’-AAGAATGGCACCCCTGTAATGT-3’ | NM_000493.3 |
|  | Reverse | 5’-ACTCCCTGAAGCCTGATCCA-3’ |  |
| *SOX9* | Forward | 5’-GGAGATGAAATCTGTTCTGGGAATG-3’ | NM_000346.3 |
|  | Reverse | 5’-TTGAAGGTTAACTGCTGGTGTTCTG-3’ |  |
| *GAPDH* | Forward | 5’-CATGTTCCAATATGATTCCAC-3’ | NM_33197.1 |
|  | Reverse | 5’-CCTGGAAGATGGTGATG-3’ |  |

**Supplementary Table 3**. Characteristics and clinical-pathological features of the 6 GC patients

| ID | Gender | Age | Histologic type | AJCC stage | Primary site | Metastasis site |
| --- | --- | --- | --- | --- | --- | --- |
| 276 | Male | 45y | Poorly differentiated adenocarcinoma | I | Stomach |  |
| 298 | Male | 67y | Poorly differentiated adenocarcinoma | I | Stomach |  |
| 335 | Female | 42y | Poorly differentiated adenocarcinoma | I | Stomach |  |
| 261 | Male | 33y | Poorly differentiated adenocarcinoma | IV | Stomach | Peritoneum |
| 318 | Male | 63y | Poorly differentiated adenocarcinoma | IV | Stomach | Peritoneum |
| 394 | Female | 71y | Poorly differentiated adenocarcinoma | IV | Stomach | Peritoneum |

**Supplementary Table 4**. Statistics of transcriptome data

| Group | | Total reads | Unique Mapped Reads | Multiple Mapped reads | Mapping Ratio |
| --- | --- | --- | --- | --- | --- |
| EGN | 276N | 69,003,304 | 50,490,103 (73.17%) | 6,277,760 (9.10%) | 82.27% |
|  | 298N | 67,096,442 | 40,382,309 (60.19%) | 14,532,118 (21.66%) | 81.84% |
|  | 335N | 69,324,948 | 44,637,081 (64.39%) | 10,294,260 (14.85%) | 79.24% |
| EGT | 276T | 71,236,116 | 57,470,732 (80.68%) | 431,952 (0.61%) | 81.28% |
|  | 298T | 72,976,500 | 59,720,982 (81.84%) | 363,688 (0.50%) | 82.33% |
|  | 335T | 71,874,696 | 60,703,833 (84.46%) | 472,216 (0.66%) | 85.11% |
| AGN | 261N | 63,224,988 | 49,850,770 (78.85%) | 3,770,128 (5.96%) | 84.81% |
|  | 318N | 72,330,254 | 46,436,766 (64.20%) | 13,802,306 (19.08%) | 83.28% |
|  | 394N | 67,887,112 | 55,012,797 (81.04%) | 911,876 (1.34%) | 82.38% |
| AGT | 261T | 65,949,614 | 47,135,460 (71.47%) | 1,845,464 (2.80%) | 74.27% |
|  | 318T | 63,374,996 | 45,978,744 (72.55%) | 3,598,026 (5.68%) | 78.23% |
|  | 394T | 66,055,610 | 52,738,291 (79.84%) | 357,036 (0.54%) | 80.38% |

EGN early-stage gastric normal tissue;

EGT early-stage gastric tumor tissue;

AGN advanced stage gastric normal tissue;

AGT advanced stage gastric tumor tissue

**Supplementary Table 5**. The 10 up and 3 down-regulated genes only in the AGC group

| Gene symbol | Cancer(FPKM) | Normal(FPKM) | F.D.R |
| --- | --- | --- | --- |
| PADI3 | 1.033333 | 0.001 | 0.045702 |
| PGBD3 | 0.62 | 0.003333 | 0.003623 |
| APOC2 | 6.666667 | 0.05 | 0.006036 |
| HOXC9 | 3.57 | 0.053333 | 0.045702 |
| KLK8 | 10.17 | 0.166667 | 0.014279 |
| DCLK3 | 0.926667 | 0.036667 | 0.046217 |
| MMP3 | 39.18667 | 2.823333 | 0.035078 |
| MMP7 | 93.71333 | 6.816667 | 0.000458 |
| CHST4 | 1.063333 | 0.08 | 0.046217 |
| NOX4 | 2.293333 | 0.18 | 0.045126 |
| KRT24 | 0.001 | 0.676667 | 0.013817 |
| MYOC | 0.096667 | 8.82 | 5.54E-06 |
| ALDOB | 28.58667 | 189.29 | 0.038452 |

**Supplementary Table 6**. The 10 up and 10 down-regulated genes only in the EGC group

| Gene symbol | Cancer(FPKM) | Normal(FPKM) | F.D.R |
| --- | --- | --- | --- |
| CA1 | 469.4633 | 0.023333 | 5.23E-05 |
| DEFA5 | 15.41667 | 0.001 | 0.000872 |
| BAAT | 13.29 | 0.001 | 1.17E-05 |
| HOXB13 | 12.17333 | 0.001 | 3.42E-07 |
| PRAC1 | 11.91333 | 0.001 | 0.027455 |
| HOXA13 | 9.31 | 0.001 | 1.30E-12 |
| APOA4 | 118.2867 | 0.013333 | 0.000802 |
| S100A7 | 8.116667 | 0.001 | 0.026077 |
| TMPRSS15 | 78.59333 | 0.01 | 0.000713 |
| OTOP3 | 7.446667 | 0.001 | 0.0001 |
| PGA4 | 9.96 | 109711.9 | 2.73E-42 |
| PGA3 | 26.57 | 288306.5 | 7.25E-51 |
| PGA5 | 3.06 | 29036.67 | 6.37E-47 |
| FGG | 0.001 | 5.72 | 1.78E-09 |
| CHIA | 0.023333 | 83.24667 | 3.03E-20 |
| GUCA1C | 0.001 | 2.616667 | 1.75E-05 |
| AQP4 | 0.03 | 24.35 | 1.74E-24 |
| ETNPPL | 0.03 | 17.64 | 3.03E-20 |
| AFM | 0.001 | 0.573333 | 0.000448 |
| ATP4B | 3.416667 | 1869.317 | 4.44E-13 |

**Supplementary Table 7**. Univariate and multivariate analyses of different prognostic factors in 103 patients with GC using Cox regression model

| Variable | All case | Univariate analysis | | Multivariate analysis | |
| --- | --- | --- | --- | --- | --- |
|  |  | HR (95% CI) | P-value | HR (95% CI) | P-value |
| Gender | | 0.889(0.394-1.780) | 0.428 |  |  |
| Male | 72 |  |  |  |  |
| Female | 31 |  |  |  |  |
| Age (years) | | 0.943(0.419-1.825) | 0.684 |  |  |
| <60 | 63 |  |  |  |  |
| ≥60 | 40 |  |  |  |  |
| Tumor size | | 2.263(1.317-4.258) | 0.016 | 1.532(0.747-3.258) | 0.253 |
| <5cm | 62 |  |  |  |  |
| ≥5cm | 41 |  |  |  |  |
| Differentiation | | 2.375(1.169-4.326) | 0.003 | 1.439(0.732-2.874) | 0.357 |
| Well | 31 |  |  |  |  |
| Moderate | 40 |  |  |  |  |
| Poor | 32 |  |  |  |  |
| Lymph node metastasis | | 0.425(0.236-0.828) | 0.003 |  |  |
| No | 43 |  |  |  |  |
| Yes | 60 |  |  |  |  |
| Serosal invasion | | 3.872(1.329-5.932) | 0.002 |  |  |
| Yes | 44 |  |  |  |  |
| No | 59 |  |  |  |  |
| AJCC stage | | 3.495(1.886-6.342) | ＜0.001 | 2.454(1.146-4.247) | 0.023 |
| I/II | 48 |  |  |  |  |
| III/IV | 55 |  |  |  |  |
| COL10A1 expression | | 3.012(1.475-6.368) | 0.003 | 2.231(1.025-5.652) | 0.026 |
| Low | 51 |  |  |  |  |
| High | 52 |  |  |  |  |

hazard ratio, HR;

95% confidence interval, 95% CI;

American Joint Committee on Cancer, AJCC

| **Supplementary Table 8**. siRNAs for SOX9 interference and COL10A1 interference | |
| --- | --- |
| siRNAs for SOX9 interference | |
| SOX9-siR-800 | 5’-GGAGACUUCUGAACGAGAGTT-3’ |
|  | 5’-CUCUCGUUCAGAAGUCUCCTT-3’ |
| SOX9-siR-1679 | 5’-CGCUCACAGUACGACUACATT-3’ |
|  | 5’-UGUAGUCGUACUGUGAGCGTT-3’ |
| SOX9-siR-2167 | 5’-GCGAAAUCAACGAGAAACUTT-3’ |
|  | 5’-AGUUUCUCGUUGAUUUCGCTT-3’ |
| SOX9-siR-NC | 5’-UUCUCCGAACGUGUCACGUTT-3’ |
|  | 5’-ACGUGACACGUUCGGAGAATT-3’ |
| siRNAs for COL10A1 interference | |
| COL10A1-siR-228 | 5’-CCUACACCAUAAAGAGUAATT-3’ |
|  | 5’-UUACUCUUUAUGGUGUAGGTT-3’ |
| COL10A1-siR-1828 | 5’-GCAACAGCAUUAUGACCCATT-3’ |
|  | 5’-UGGGUCAUAAUGCUGUUGCTT-3’ |
| COL10A1-siR-2013 | 5’-CCAUCAUCGAUCUCACAGATT-3’ |
|  | 5’-UCUGUGAGAUCGAUGAUGGTT-3’ |
| COL10A1-siR-NC | 5’-UUCUCCGAACGUGUCACGUTT-3’ |
|  | 5’-ACGUGACACGUUCGGAGAATT-3’ |

**Supplementary Table 9**. Correlations between COL10A1 and SOX9 protein levels and the clinical-pathological parameters of GC patients

| Variables | Expression of COL10A1 | | | Expression of SOX9 | | |
| --- | --- | --- | --- | --- | --- | --- |
|  | Low expression(%) | High expression(%) | p | Low expression(%) | High expression(%) | p |
| Gender | | | | | | |
| Male | 36(57.1) | 27(42.9) | 0.351 | 37(58.7) | 26(41.3) | 0.170 |
| Female | 24(66.7) | 12(33.3) |  | 16(44.4) | 20(55.6) |  |
| Age(years) | | | | | | |
| <60 | 21(63.6) | 12(36.4) | 0.663 | 16(48.5) | 17(51.5) | 0.476 |
| ≥60 | 39(59.1) | 27(40.9) |  | 37(56.1) | 29(43.9) |  |
| Tumor size | | | | | | |
| <5cm | 29(74.4) | 10(25.6) | 0.016 | 26(66.7) | 13(33.3) | 0.023 |
| ≥5cm | 29(50.0) | 29(50.0) |  | 25(43.1) | 33(56.9) |  |
| Differentiation | | | | | | |
| Well | 36(76.6) | 11(23.4) | 0.005 | 32(68.1) | 15(31.9) | 0.022 |
| Moderate | 19(52.8) | 17(47.2) |  | 16(44.4) | 20(55.6) |  |
| Poor | 5(33.3) | 9(66.7) |  | 5(33.3) | 10(66.7) |  |
| Lymph node metastasis | | | | | | |
| No | 22(84.6) | 4(15.4) | 0.004 | 21(80.8) | 5(43.8) | 0.001 |
| Yes | 38(52.1) | 35(47.9) |  | 32(19.2) | 41(56.2) |  |
| Serosal invasion | | | | | | |
| No | 54(69.2) | 24(30.8) | 0.001 | 47(60.3) | 31(39.7) | 0.015 |
| Yes | 6(30.0) | 14(70.0) |  | 6(30.0) | 14(70.0) |  |
| AJCC stage | | | | | | |
| I/II | 33(76.7) | 10(23.3) | 0.004 | 31(72.1) | 12(27.9) | 0.001 |
| III/IV | 27(48.2) | 29(51.8) |  | 22(39.3) | 34(60.7) |  |

American Joint Committee on Cancer, AJCC

**Supplementary Table 10**. Univariate and multivariate analyses of different prognostic factors in 99 patients with GC using Cox regression model

| Variable | All case | Univariate analysis | | Multivariate analysis | |
| --- | --- | --- | --- | --- | --- |
|  |  | HR (95% CI) | P-value | HR (95% CI) | P-value |
| Gender | | 0.621(0.249-1.551) | 0.308 |  |  |
| Male | 63 |  |  |  |  |
| Female | 36 |  |  |  |  |
| Age (years) | | 1.150(0.470-2.812) | 0.759 |  |  |
| <60 | 33 |  |  |  |  |
| ≥60 | 66 |  |  |  |  |
| Tumor size | | 1.727(1.483-2.093) | 0.026 | 1.964(0.813-4.745) | 0.134 |
| <5cm | 58 |  |  |  |  |
| ≥5cm | 39 |  |  |  |  |
| Differentiation | | 1.949(1.276-2.976) | 0.002 | 0.930(0.516-1.677) | 0.810 |
| Well | 48 |  |  |  |  |
| Moderate | 36 |  |  |  |  |
| Poor | 14 |  |  |  |  |
| Lymph node metastasis | | 0.351(0.173-0.713) | 0.004 |  |  |
| No | 26 |  |  |  |  |
| Yes | 73 |  |  |  |  |
| Serosal invasion | | 2.596(1.371-4.916) | 0.003 |  |  |
| Yes | 20 |  |  |  |  |
| No | 78 |  |  |  |  |
| AJCC stage | | 2.972(1.287-5.775) | 0.005 | 2.023(1.046-3.912) | 0.036 |
| I/II | 43 |  |  |  |  |
| III/IV | 56 |  |  |  |  |
| COL10A1 expression | | 2.229(1.224-4.058) | 0.009 | 2.586(1.336-4.022) | 0.024 |
| Low | 60 |  |  |  |  |
| High | 39 |  |  |  |  |
| SOX9 expression | | 2.819(1.557-5.107) | 0.001 | 1.567(1.483-3.093) | 0.031 |
| Low | 53 |  |  |  |  |
| High | 46 |  |  |  |  |

hazard ratio, HR;

95% confidence interval, 95%CI;

American Joint Committee on Cancer, AJCC
