# Supplementary figures and images for: TGF-β1-SOX9 axis-inducible COL10A1 promotes invasion and metastasis in gastric cancer via epithelial-to-mesenchymal transition
Source: Cell Death Dis. 2018 Aug 28;9(9):849. doi: 10.1038/s41419-018-0877-2 (PMC6113209; doi:10.1038/s41419-018-0877-2)

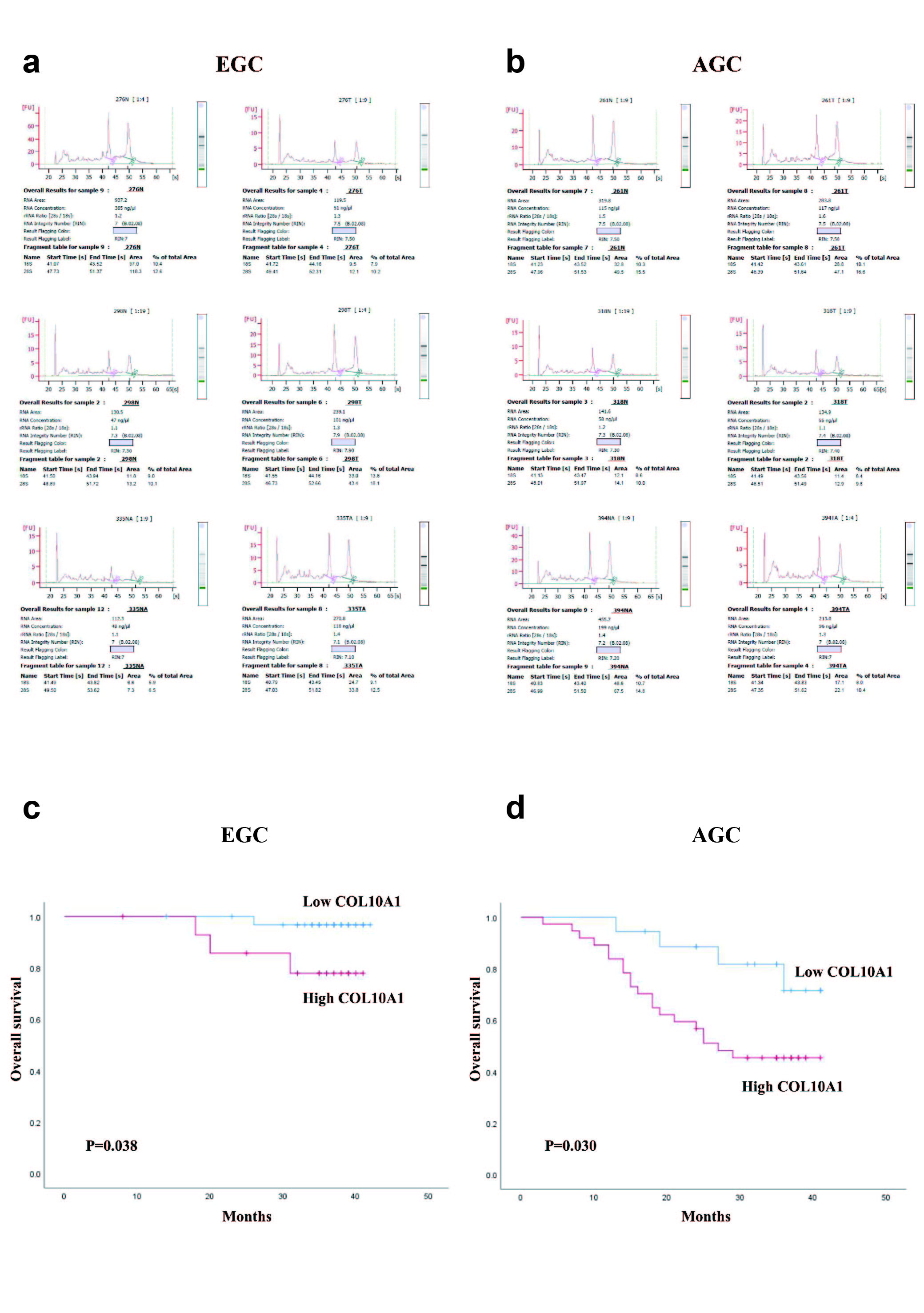

Supplement: Supplementary file 2 — Supplementary Figure 1 [file 41419_2018_877_MOESM2_ESM.jpg]

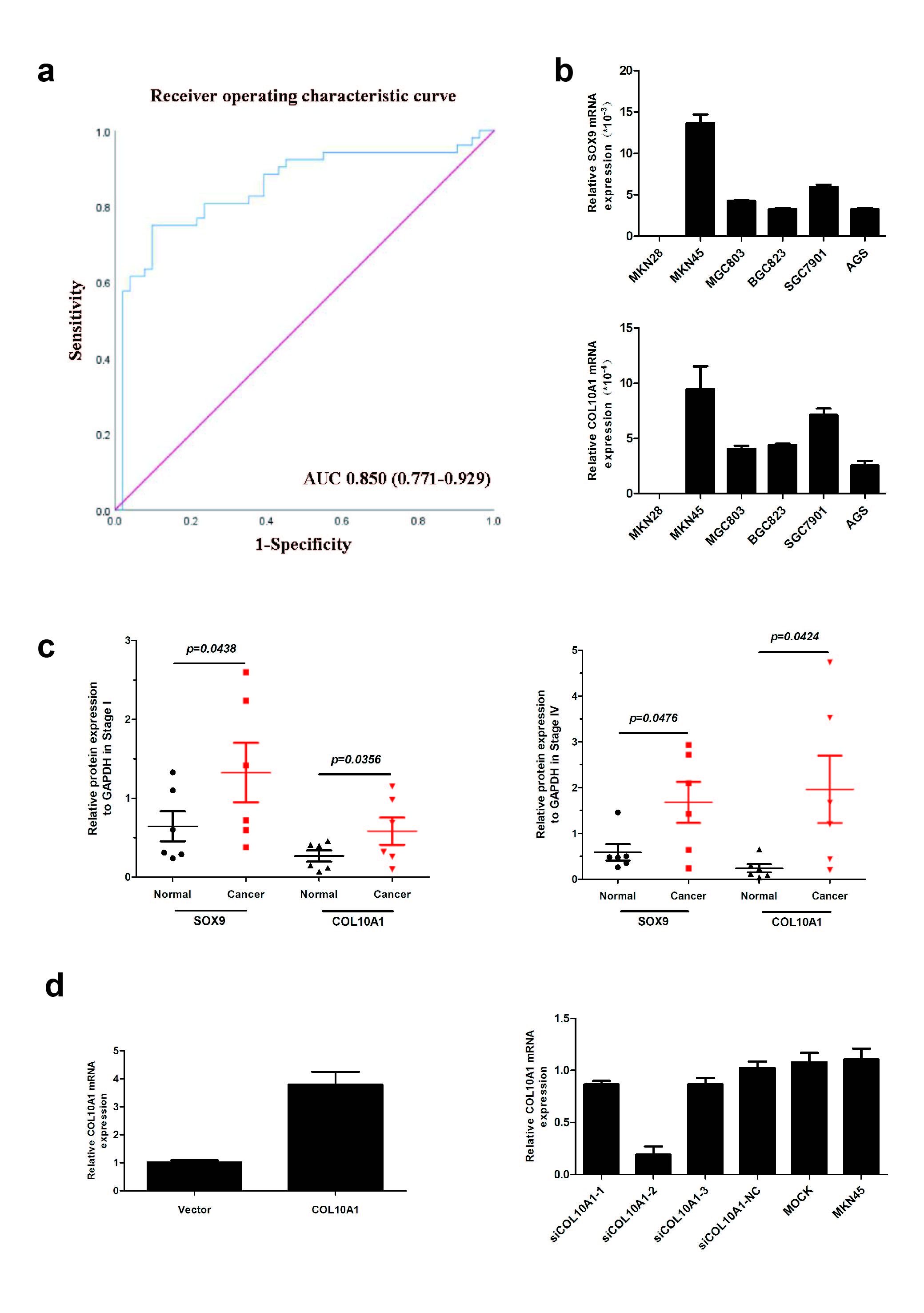

Supplement: Supplementary file 3 — Supplementary Figure 2 [file 41419_2018_877_MOESM3_ESM.jpg]

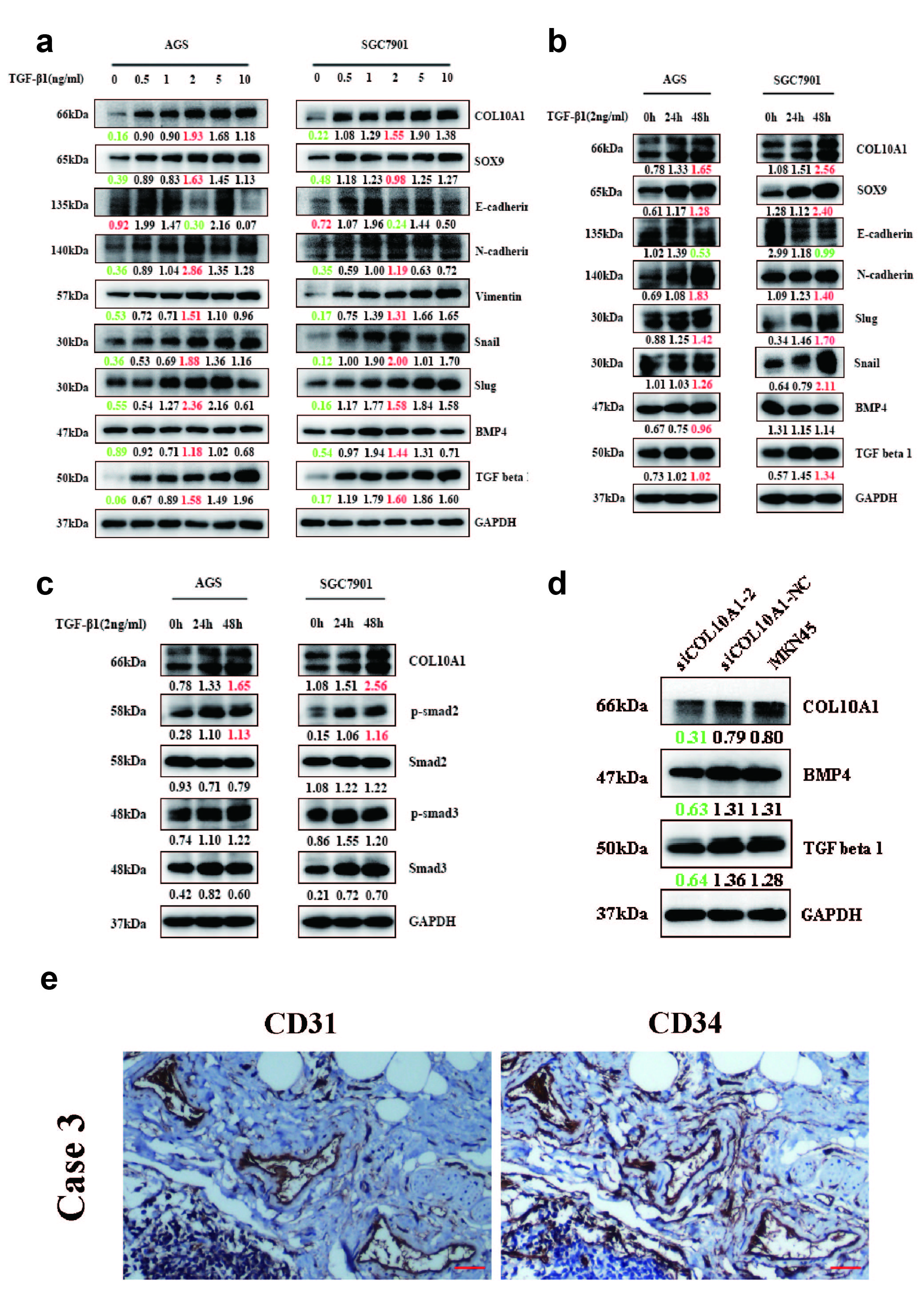

Supplement: Supplementary file 4 — Supplementary Figure 3 [file 41419_2018_877_MOESM4_ESM.jpg]

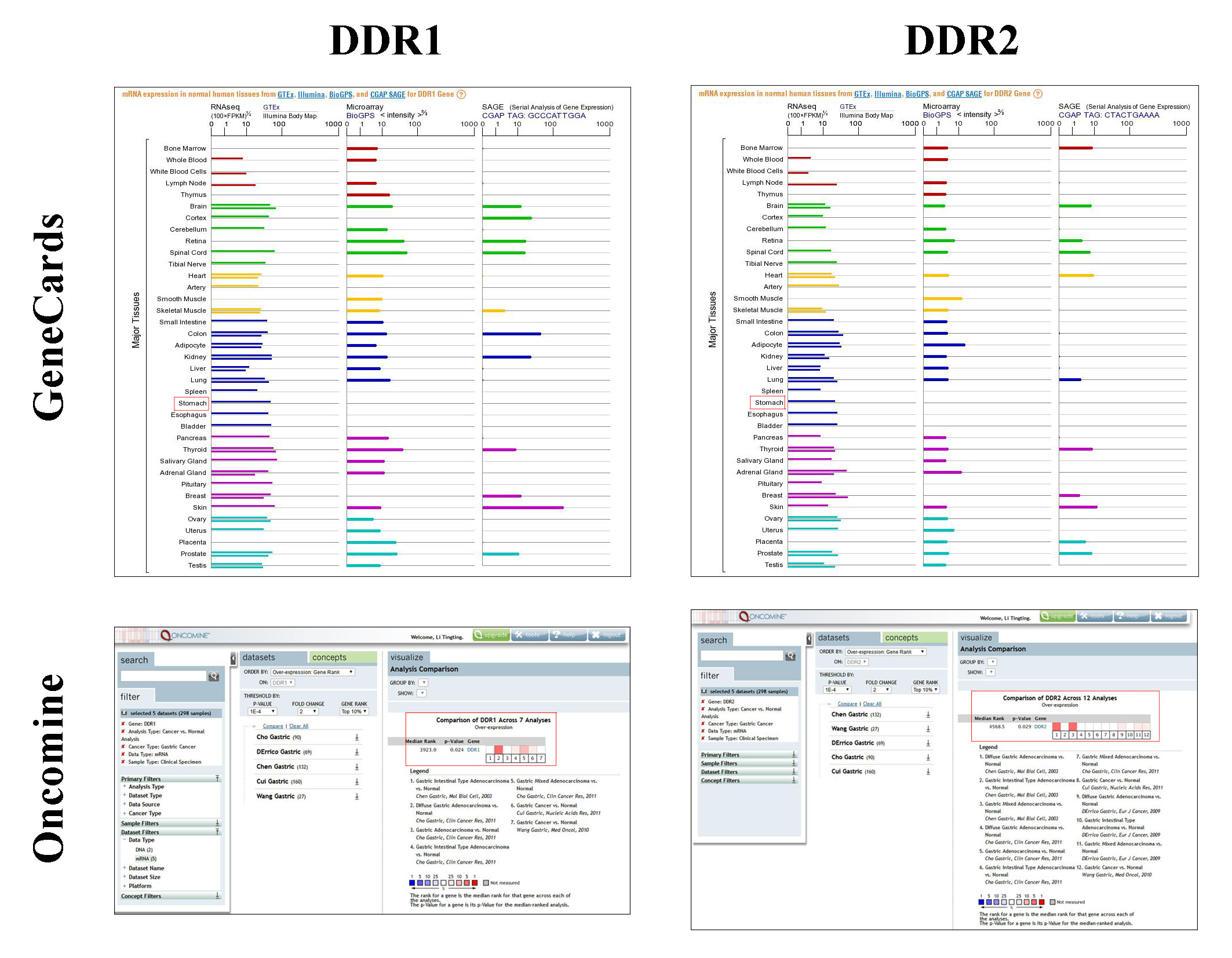

Supplement: Supplementary file 5 — Supplementary Figure 4 [file 41419_2018_877_MOESM5_ESM.jpg]
