# Supplementary material for: TGF-β1-SOX9 axis-inducible COL10A1 promotes invasion and metastasis in gastric cancer via epithelial-to-mesenchymal transition
Source: Cell Death Dis. 2018 Aug 28;9(9):849. doi: 10.1038/s41419-018-0877-2 (PMC6113209; doi:10.1038/s41419-018-0877-2)
Supplement: Supplementary file 6 — Supplementary figure legends [file 41419_2018_877_MOESM6_ESM.docx]

**Supplementary Figure 1** The quality of RNA for RNAseq analysis and the prognostic value of COL10A1 mRNA in OS. (**a** and **b**) The quality of RNA of the six patients’ samples profiled for RNAseq analysis in EGC and AGC groups. (**c** and **d**) The prognostic value of COL10A1 mRNA in OS in EGC and AGC groups.

**Supplementary Figure 2** The mRNA and protein expressions of SOX9 and COL10A1 in GC cell lines. (**a**) ROC analysis was used to calculate the cut-off value of high or low COL10A1 mRNA levels. (**b**) The mRNA expression levels of SOX9 and COL10A1 were detected in GC cell lines by QPCR. (**c**) SOX9 and COL10A1 protein expression levels in normal and GC tissues of Stage I and Stage IV. (**d**) The mRNA expression levels of COL10A1 were detected using QPCR by transfection of SGC7901 cells with COL10A1-sense plasmids and MKN45 cells with COL10A1-siRNA.

**Supplementary Figure 3** The TGF-β1-SOX9-axis promotes COL10A1-mediated EMT. (**a**, **b** and **c**) Proteins extracted from AGS and SGC7901 cells treated with various doses of TGF-β1 or various times at 2ng/ml TGF-β1 were used to detect EMT biomarkers, Smad2, Smad3, phosphorylated smad2 and smad3 using western blot analysis. (**d**) BMP4 and TGF-β1 were detected after transfection of MKN45 cells with COL10A1-siRNAusing western blot analysis. (**e**) CD31 and CD34 expression levels in peritoneal metastatic specimens were detected using IHC analysis. All results shown were reproduced in at least three independent experiments.

**Supplementary Figure 4** A search of DDR1/2 markers in GC using GeneCards and Oncomine databases.
